# An International Randomised Placebo-Controlled Trial of a Four-Component Combination Pill (“Polypill”) in People with Raised Cardiovascular Risk

**DOI:** 10.1371/journal.pone.0019857

**Published:** 2011-05-25

**Authors:** PILL Collaborative Group

**Affiliations:** University of British Columbia, Canada

## Abstract

**Background:**

There has been widespread interest in the potential of combination cardiovascular medications containing aspirin and agents to lower blood pressure and cholesterol (‘polypills’) to reduce cardiovascular disease. However, no reliable placebo-controlled data are available on both efficacy and tolerability.

**Methods:**

We conducted a randomised, double-blind placebo-controlled trial of a polypill (containing aspirin 75 mg, lisinopril 10 mg, hydrochlorothiazide 12.5 mg and simvastatin 20 mg) in 378 individuals without an indication for any component of the polypill, but who had an estimated 5-year cardiovascular disease risk over 7.5%. The primary outcomes were systolic blood pressure (SBP), LDL-cholesterol and tolerability (proportion discontinued randomised therapy) at 12 weeks follow-up.

**Findings:**

At baseline, mean BP was 134/81 mmHg and mean LDL-cholesterol was 3.7 mmol/L. Over 12 weeks, polypill treatment reduced SBP by 9.9 (95% CI: 7.7 to 12.1) mmHg and LDL-cholesterol by 0.8 (95% CI 0.6 to 0.9) mmol/L. The discontinuation rates in the polypill group compared to placebo were 23% vs 18% (RR 1.33, 95% CI 0.89 to 2.00, p = 0.2). There was an excess of side effects known to the component medicines (58% vs 42%, p = 0.001), which was mostly apparent within a few weeks, and usually did not warrant cessation of trial treatment.

**Conclusions:**

This polypill achieved sizeable reductions in SBP and LDL-cholesterol but caused side effects in about 1 in 6 people. The halving in predicted cardiovascular risk is moderately lower than previous estimates and the side effect rate is moderately higher. Nonetheless, substantial net benefits would be expected among patients at high risk.

**Trial Registration:**

Australian New Zealand Clinical Trials Registry ACTRN12607000099426

## Introduction

In 2001, the World Health Organisation and The Wellcome Trust convened a meeting of experts to discuss evidence-based and affordable interventions for non-communicable diseases.[1] A major impetus for the meeting was the potential of fixed-dose combination pills containing aspirin, statin and blood pressure lowering agents, noting “the use of a single pill could well encourage patients to adhere to treatment as well as seriously reduce the cost of the drugs.” A programme of research was outlined, including stability and bio-availability testing followed by assessment of short-term effects on blood pressure, cholesterol, platelet aggregation, safety and side effects, ideally including developing country participants. In 2002, the WHO Annual Report outlined the substantial potential public health impact and cost-effectiveness of scaling up access to combination treatment.[2] An editorial that year also noted that a four component combination pill would be expected to reduce cardiovascular risk by about 75% among people with vascular disease.[3] In 2003 the first full exposition of the scientific evidence for cardiovascular combination pills was published in the medical literature.[4], [5], [6], [7] The ‘polypill’ term was coined and gained widespread attention, in large part due to the recommendation to treat everyone aged over 55 years in developed countries. The proposal to target treatments based on age alone has been highly polarizing. An alternate approach, now recommended by major cross-disciplinary guidelines[8], [9], [10], [11] and the European Medicines Agency (EMA),[12] is to target treatments principally on the basis of global cardiovascular risk. As noted by the EMA[12] “the terms primary/secondary prevention have yielded their place for a more comprehensive strategy aimed at treating patients at high risk of cardiovascular disease…current therapeutic strategies are aimed at identifying global cardiovascular disease risk in an individual and treating all risk factors. Global risk intervention, rather than single risk modification is the standard of care”.

We therefore set out to conduct the trial recommended by the meeting of the WHO and The Wellcome Trust, assessing short-term efficacy and side effects, among people at raised global cardiovascular risk. The trial aimed to assess the full effects of polypill treatment compared to placebo. Such information would be relevant to research among people with raised cardiovascular risk (many of whom are not currently treated as they do not have ‘hypertension’ or ‘dyslipidaemia’) and we planned this initiative as a necessary first step before starting a large long-term trial in this population. The trial was also planned to inform research and treatment in people with established vascular disease, since the risk factor reductions would be generalisable but use of placebo is not appropriate in this group.

## Methods

The protocol for this trial and supporting CONSORT checklist are available as supporting information; see Checklist S1 and Protocol S1.

We conducted a randomised controlled trial in seven countries – Australia (n = 21), Brazil (n = 8), India (n = 109), Netherlands (n = 102), New Zealand (n = 12), United Kingdom (n = 113) and United States (n = 13). Approval for the trial was obtained from the institutional ethics committee of each centre and all participants provided written informed consent. The trial is registered with the Australian New Zealand Clinical Trials Registry (ACTRN 12607000099426).

### Participants

The key eligibility criteria were raised cardiovascular risk together with no indication for or contraindication to treatment with component medicines in the polypill. Individuals were included if they were adults (≥18 years) with a cardiovascular disease (CVD) risk over 5 years of at least 7.5%, determined by the Framingham risk function[13] using data on age, gender, blood pressure, total cholesterol, HDL cholesterol, diabetes status and cigarette smoking status (left ventricular hypertrophy was assumed to be absent for the purpose of CVD risk calculation). A value of 7.5% on Framingham function was chosen as half the threshold value above which all modalities are recommended in the first set of guidelines based on absolute risk.[14] While Framingham performs well in modern clinical practice after calibration[15] it is nonetheless imperfect.[16] For example, it does not incorporate some risk factors that have additional predictive value. Therefore, those with an estimated 5-year risk of 5.0– <7.5% from the Framingham function were also eligible if two or more additional risk factors were present: body mass index (BMI) >30 kg/m^2^; waist circumference >102 cm in men or >88 cm in women; heart rate >80 beats/min; fasting glucose 5.6– <7 mmol/L; triglycerides >1.7 mmol/L; family history of premature coronary heart disease (CHD) or ischaemic stroke in a first degree male relative before the age of 55 years or a first degree female relative before the age of 65 years; or glomerular filtration rate (GFR) <60 mL/min. Uniform definitions were used for all centres. To be included, the participants had to have no contraindication to treatment with low-dose aspirin, angiotensin-converting enzyme (ACE) inhibitor, low-dose diuretic or statin; nor any indication or recommendation under local guidance for treatment with any of these medicines. The participating countries varied in their extent of risk factor threshold-based (eg. hypertension treatment) or absolute risk-based treatment practices. Therefore some participants had comparatively high risk factor levels (but moderate absolute risk), while others had comparatively high absolute risk (but moderate risk factor levels). Participants taking other antiplatelet, blood pressure lowering or cholesterol lowering medicines were also excluded, as were patients with diabetes mellitus or GFR ≤30 ml/min/1.73 m^2^.

### Randomisation, Allocation Concealment and Study Interventions

Eligible participants were randomised to the Red Heart Pill (RHP, a polypill comprising a bilayered tablet containing aspirin 75 mg, lisinopril 10 mg, hydrochlorothiazide 12.5 mg and simvastatin 20 mg) or an identical placebo, in a 1:1 ratio. Participants, research staff and and co-ordinating centre staff were all blinded to the allocation. Study treatment was taken once a day in the evening with food. There was no ‘run-in’ period. Study treatments were allocated using a central computer-based randomisation service at The Clinical Trials Research Unit, University of Auckland, accessible by internet, using a minimisation algorithm including age, sex and centre. Participants were recruited from 17 October 2008 to 22 December 2009. Regulatory delays in importing trial treatment were prolonged and recruitment was 22 participants less than intended, since the study medication expiry date was reached.

### Concomitant Interventions

The use of concomitant open-label therapy was allowed at the discretion of the responsible clinician. Without the need to unblind, additional treatment with open-label therapy was permitted –75 mg aspirin; any beta-blocker, calcium channel blocker, angiotensin receptor blocker or alpha-blocker; 10–20 mg lisinopril and/or 12.5 mg hydrochlorothiazide or 2.5 mg bendrofluazide; 10–20 mg simvastatin – if any of these treatments became indicated during the trial. If there was a need for higher doses of aspirin, ACE inhibitor, diuretic or simvastatin, these were provided as open label treatment and the trial treatment was stopped. Open-label fibrate (with the exception of gemfibrozil) could also be added, without the need to unblind or stop the trial treatment, provided that appropriate monitoring for rhabdomyolysis was instituted.

### Study Procedures

Participants were seen at 2, 6 and 12 weeks after randomisation, with a post-study follow-up appointment 4 weeks after the final 12-week visit. At study visits, information on adherence to and tolerability of study treatments, blood pressure, lipids and occurrence of adverse events was obtained. Blood pressure was recorded as the mean of two measurements made after the patient was rested for at least 5 minutes in the seated position, using a standardised automated sphygmomanometer that had been validated according to the protocol of the Association for the Advancement of Medical Instrumentation (AAMI) or British Hypertension Society, International protocol version. Lipid measurements were undertaken at local laboratories holding ISO 15189 (2003 or later) accreditation. The trial was co-ordinated by The Clinical Trials Research Unit, at The University of Auckland which provided an internet based clinical trial management system. An independent monitor completed bi- monthly site visits to ensure the trial was conducted according to the protocol, good clinical practice guidelines and relevant local regulatory requirements. All participants provided informed consent.

### Outcomes

The primary study outcomes were change in systolic blood pressure (SBP), change in LDL-cholesterol and tolerability (proportion who withdrew from trial treatment for any reason). Secondary outcomes were treatment adherence (% of prescribed treatment according to pill counts, with participants asked to return all used blisters and unused trial treatment to study visits), diastolic blood pressure, total cholesterol, HDL cholesterol, total cholesterol:HDL cholesterol ratio, non-HDL cholesterol, triglycerides, frequency of switching/adding open-label treatment and estimated effects on CVD risk.

### Sample Size

It was estimated that 400 participants would provide 85% power at 2p = 0.05 to detect a 0.25 mmol/l difference in LDL-cholesterol and 80% power to detect a 4 mmHg difference in systolic blood pressure between the intervention and control groups, assuming standard deviations around the change from baseline levels of 0.8 mmol/l and 14 mmHg respectively, and a 10% absolute difference in tolerability. This sample size would also provide a 95% confidence interval width of about 6 mmHg and 0.3 mmol/L for estimates of SBP and LDL-cholesterol reductions respectively.

### Statistical analysis

Primary analysis was by intention-to-treat. Means of changes in blood pressure and lipid values from baseline to 12 weeks between polypill and placebo groups were compared using a 2 sample t-test. Adjusted analyses were carried out by including the stratification factors in an analysis of covariance regression model with change in blood pressure and lipid variable as the dependent variable. Last observation carried forward was used for missing data at 12 weeks, with a sensitivity analysis also based on repeated measures using a mixed models approach to the analysis of covariance. The proportions that withdrew from trial treatment (tolerability) at 12 weeks between polypill and placebo groups were compared using the chi-squared test, with those without follow-up information assumed to have stopped study treatment. It was determined after trial completion that there had been mislabeling of a sequence of treatment packs that affected 14 participants who received active treatment rather than placebo. Therefore an additional sensitivity analysis was conducted excluding these participants. All analyses were done using SAS [version 9.1.3].

Expected reductions in cardiovascular risk were estimated using data from systematic reviews, which have shown that each medication class confers approximately similar proportional reductions in cause-specific outcomes across a wide range of patient populations, with no major differences between agents (after accounting for the extent of risk factor reduction for SBP and LDL) and even when event rates vary tenfold or more.[17], [18], [19], [20], [21] For example, aspirin produces about a one-fifth reduction in CHD and ischaemic stroke risk in ‘primary’ and ‘secondary’ prevention.[20] There is clear evidence that the proportional reductions in major outcomes achieved with each treatment modality are approximately the same in the presence or absence of other interventions[17], [19], [20], [22] (which is expected given the lack of interaction between treatments in terms of risk factor reduction[23] and the epidemiology of blood pressure and cholesterol joint effects[24], [25]). Therefore, the combined effects are best estimated by multiplying relative risks together, after adjusting for the size of SBP and LDL-cholesterol reductions. Thus for example, since 1 mmol/L LDL-cholesterol reduction, 10 mmHg SBP reduction and aspirin each individually lower CHD risk by 42%[5], 22%[17] and 20%[20] respectively (ie. RRs are 0.58, 0.78 and 0.80 respectively), the expected joint effects of a 0.5 mmol/L LDL-cholesterol reduction, 5 mmHg SBP reduction and aspirin would be approximately a 46% lower CHD risk (since 0.58^0.5/1.0^×0.78^5/10^×0.80 = 0.54, and (1−0.54) ×100%  = 46%). Combining the proportional effects with data on current event rates (rather than event rates in trials, which are often out of date and not representative), provides the best estimates of expected absolute treatment effects.[26], [27]

## Results

**Figure 1:**
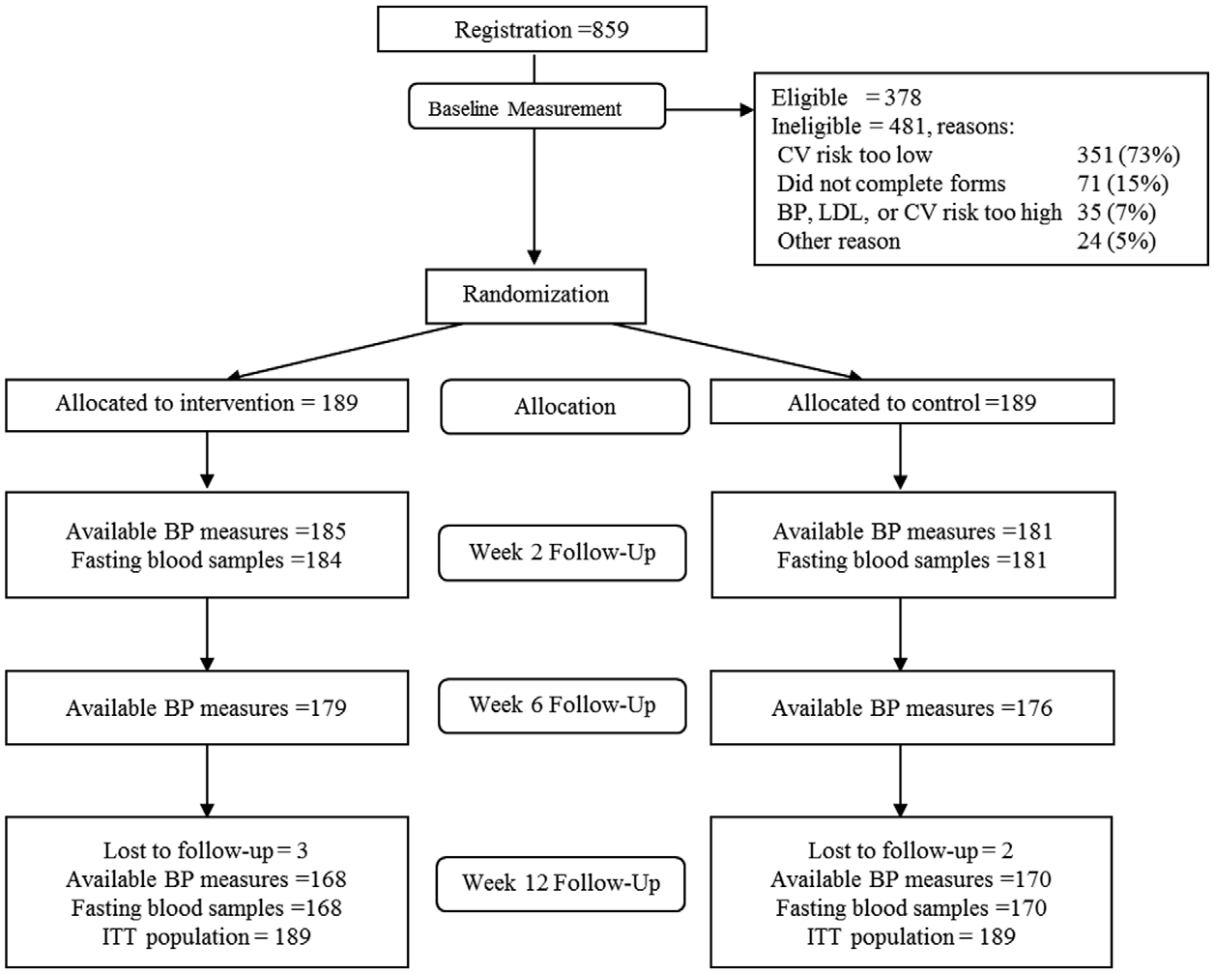
CONSORT flow chart. This figure shows the flow of patients through the trial according to the criteria recommended in the CONSORT Guidelines.

**Figure 2:**
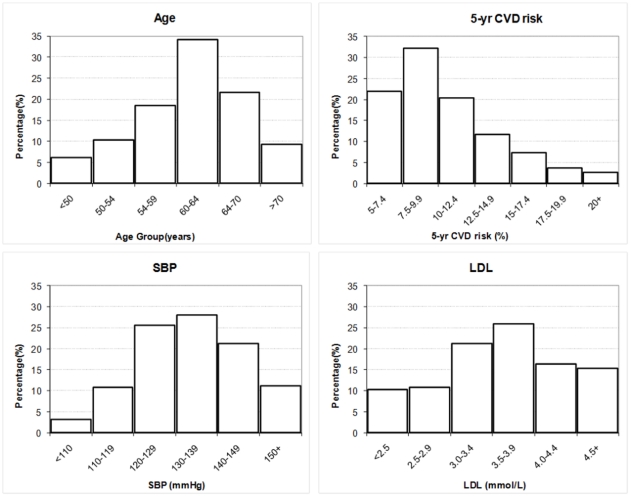
Baseline frequency distributions of age, LDL-cholesterol, SBP and 5-year cardiovascular risk. This figure shows the frequency distribution of participants according to their baseline levels of age, LDL-cholesterol, SBP and 5-year cardiovascular risk.

A total of 378 participants were randomised into the study (Figure 1) from 17 October 2008 to 22 December 2009. At 12 weeks, vital status was available for 373 (98.7%) of participants and data on SBP and LDL-cholesterol levels were available for 338 (89.4%). There was good balance between randomised groups across a range of characteristics at study entry (Table 1). The frequency distributions for age, SBP, LDL-cholesterol and estimated 5-year cardiovascular risk are shown in Figure 2. As can be seen, most patients were aged between 50 and 70 years and there was a wide range of baseline SBP and LDL-cholesterol levels; for example according to JNC 7 criteria[28] 33% would be regarded as having ‘hypertension’ with SBP >140 mmHg, 52% ‘pre-hypertension’ with SBP 120–139, and 14% having ‘normal’ blood pressure of SBP <120 mmHg. Overall 22% of participants had a 5-year cardiovascular risk of 5–7.5% by the Framingham function (all of whom had two or more other risk factors, see Methods), and 3% had 5-year cardiovascular risk over 20% (ie. equivalent to the risk faced by those with previous vascular disease events[29]).

**Table 1:** Baseline characteristics.

	Red Heart Pilln = 189		Placebon = 189	
*Cardiovascular risk factors in Framingham score*				
Age (yrs)	61.2	(7.2)	61.6	(7.2)
Male	153	(81%)	152	(80%)
Blood pressure (mmHg)	132/80	(13/9)	136/81	(14/9)
LDL-cholesterol (mmol/L)	3.7	(0.9)	3.6	(0.9)
Total cholesterol (mmol/L)	5.6	(1.1)	5.4	(1.0)
HDL (mmol/L)	1.2	(0.3)	1.3	(0.4)
Smoker (or quit within the last year)	79	(42%)	74	(39%)
*Other cardiovascular risk factors* *				
Body mass index >30 kg/m^2^, waist circumference >102 cm in men or >88 cm in women	88	(47%)	90	(48%)
Heart rate >80 beats/min	48	(25%)	46	(24%)
Fasting glucose 5.6– <7 mmol/L	55	(29%)	60	(32%)
Family history of premature coronary heart disease or ischaemic stroke	87	(46%)	77	(41%)
Triglycerides >1.7 mmol/L	69	(37%)	53	(28%)
Glomerular filtration rate (GFR) <60 mL/min	18	(10%)	23	(12%)
At least 2 of the above*	120	(63%)	117	(62%)
*Cardiovascular risk*				
5-year cardiovascular risk - Framingham function	10%	(4.1%)	11%	(4.5%)
10-yr fatal cardiovascular risk – SCORE function	4.3%	(5.0%)	4.9%	(5.4%)
*Medications*				
Prescribed or over-the-counter medicines	59	(31%)	43	(23%)
Vitamin and/or mineral capsules/tablets	43	(23%)	37	(20%)
Other dietary supplements	34	(18%)	31	(16%)
Any other complementary or alternative medicine	5	(3%)	7	(4%)
*Current lifestyle factors*				
Moderate physical exercise in last 7 days (mins)	211	(240)	256	(279)
Vigorous physical exercise in last 7 days (mins)	23	(104)	16	(49)
Formal exercise programme	4	(2%)	5	(3%)
Seeing a dietician or other nutritional counsellor or on a weight control programme	1	(1%)	1	(1%)
Smoking cessation programme	4	(2%)	2	(1%)
*Other*				
Currently drink alcohol once a week or more (on most weeks for at least the last year)	132	(69%)	142	(75%)

Data are mean (sd) or n (%).

*Participants with Framingham 5-yr CVD risk 5–7.5% were eligible for the trial if they had at least two such factors.

### Effects on blood pressure and cholesterol levels

**Figure 3:**
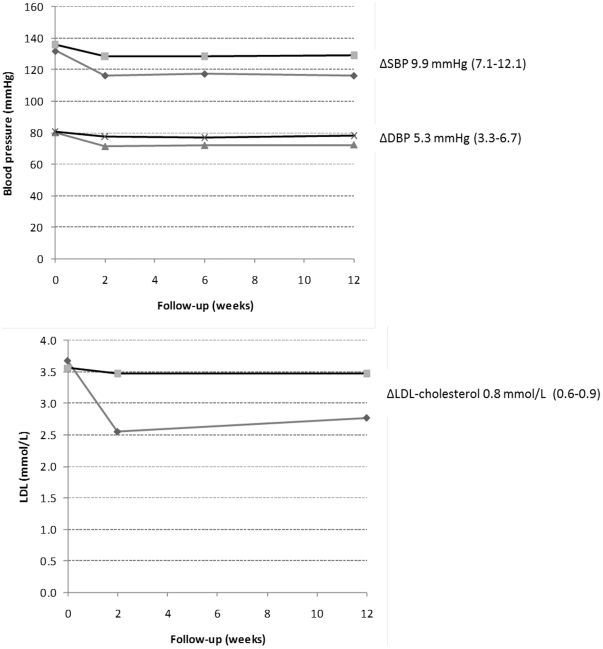
Blood pressure and LDL-cholesterol changes. This figure shows the changes in blood pressure and LDL-cholesterol over the 12 week trial period, according to active (dark line) or placebo (grey line).

Over the duration of follow-up, SBP was reduced by an average of 9.9 (95% CI: 7.7 to 12.1) mmHg compared to the placebo group, while LDL-cholesterol was reduced by an average of 0.8 (95% CI: 0.6 to 0.9) mmol/L (both p<0.0001, see Figure 3). Treatment differences were achieved at two weeks and maintained throughout follow-up. Overall effect estimates were not importantly altered by adjusted analyses or by the exclusion of participants receiving mislabeled treatment packs (SBP reduction 10.4, 95% CI 8.1 to 12.7 mmHg and LDL-cholesterol reduction 0.8, 95% CI 0.7 to 0.9 mmol/L).

There was also a reduction in DBP of 5.3 mmHg (95% CI 3.9 to 6.7, p<<0.001), in total cholesterol of 0.8 mmol/L (95%CI, 0.7–1.0, p<0.001) and in triglycerides of 0.2 mmol/L (95%CI 0.1−0.3, p = 0.001). There was no clear effect on HDL (0.02 mmol/L increase, 95% CI −0.04 to 0.04, p = 0.9).

### Tolerability and side effects

Overall through the 12 weeks, 44 (23%) in the polypill group compared to 33 (18%) in the placebo group discontinued treatment (RR 1.33, 95% CI 0.89 to 2.00 p = 0.2). Most discontinuations occurred early: 29 (66%) of the 44 discontinuations in the polypill group occurred by week 6. The main reasons for discontinuation of trial treatment by randomised group are shown in Table 2.

**Table 2:** Main reasons for stopping study treatment and side effects.

	Reported side effects of sufficient severity to discontinue study treatment*					Reported side effects not necessitating discontinuation of study treatment				
	Red Heart Pill		Placebo		P-value	Red Heart Pill		Placebo		P-value
	n	%	n	%		n	%	n	%	
Gastric irritation	6	3%	1	1%	0.06	23	12%	6	3%	0.0005
Increased bleeding tendency	0	0%	0	0%		4	2%	1	1%	0.2
Cough	3	2%	2	1%	0.7	19	10%	3	2%	0.0002
Light headed/dizziness/hypotension	7	4%	2	1%	0.09	28	15%	8	4%	0.0002
Muscle pain or weakness	1	1%	2	1%	0.6	13	7%	14	7%	0.9
Headache	1	0%	0	0%		4	2%	3	2%	0.6
Diarrhoea	0	0%	0	0%		4	2%	5	3%	0.8
Fatigue	3	2%	2	1%	0.7	13	7%	10	5%	0.4
Abdominal pain	0	0%	0	0%		4	2%	1	1%	0.2
Constipation	0	0%	0	0%		10	5%	4	2%	0.08
Flatulence	0	0%	0	0%		6	3%	5	3%	0.7
Other side effect	13	6%	12	6%	0.8	39	21%	28	15%	0.07
Patient choice	0	0%	3	2%	0.08					
Total**	34	18%	24	13%	0.2	81	43%	59	31%	0.003

*participants without relevant follow-up data at 12 weeks (10 vs 9) were assumed to have stopped treatment in the definition of tolerability as the primary trial outcome, which was therefore 44 (23%) vs 33 (18%).

**for patients discontinuing treatment, the total is a direct sum as data reflect the main reason for stopping for each patient. For patients not discontinuing treatment, the total refers to the number of people reporting one or more side effects.

A total of 110 (58%) of the polypill group and 79 (42%) of the control group reported side effects (p = 0.001). Most side effects did not necessitate stopping treatment. The excesses were mainly attributable to the well known side effects of aspirin [gastric irritation and/or bleeding tendency occurring in 32 (17%) of the polypill group and 11 (6%) of the placebo group] and of ACE inhibitor-based blood pressure lowering [cough and/or light headedness, dizziness or hypotension occurring in 57 (30%) of the polypill group and 20 (11%) of the placebo group]. Most side effects were apparent early on: at week 2, side effects were reported by 41% vs 26% (77 vs 49 people), whereas only 14% vs 11% (26 vs 20) reported new side effects in week 6 and only 4% vs 5% (7 vs 10) reported new side effects at week 12. A total of 353 participants answered the question “what trial treatment do you think you have been taking?” at the end of follow-up. The answer was correct for 79% (139/177) of people allocated polypill and for 59% (104/176) of people allocated placebo (p<0.0001 for difference).

Eight serious adverse events were reported, four in each group (polypill group - chest pain, newly diagnosed Type 2 diabetes, removal of wisdom teeth, syncope; placebo group – syncope, depression, transient ischaemic attack, hip fracture). There were no deaths, major vascular events, major bleeds or episodes of gastrointestinal ulceration.

Overall, the proportion of scheduled treatment taken according to self-reported pill counts was 82% for the polypill group and 86% for the placebo group (p = 0.1). Open-label therapy was required infrequently during follow-up: for blood pressure lowering (4 vs 3 participants), cholesterol lowering (0 vs 4) and antiplatelet therapy (2 vs 3).

### Predicted effects on cardiovascular risk

The estimated effects on cardiovascular events and other major outcomes for those continuing treatment long-term are shown in Table 3. One would expect an approximate 60% reduction in CHD and ischaemic stroke risk, little overall effect on haemorrhagic stroke risk (the beneficial effects of blood pressure lowering balancing out the adverse effects of aspirin) and a 50% increase in the risk of extra-cranial bleeding. The net effects of such treatment on any major outcome thus importantly depend on the event rates of each component outcome. In a patient group at similar risk to the average in this trial one would expect more than a halving in CVD events and about a halving in any major event (stroke, CHD or major bleed). Over 5 years of treatment, about 1 in 18 would benefit in terms of avoiding a major event, with the large majority of the net benefit due to the SBP and LDL-reduction. Among untreated individuals with a history of coronary artery disease, event rates are higher, particularly for CHD and ischaemic stroke.[29], [30] Hence compared to a lower-risk population, the proportional reductions for the composite of any major event are a little greater and the absolute benefits are much greater, being of clear clinical importance for each component. Overall about 1 in 4 high risk people would be predicted to avoid a major event over 5 years.

**Table 3:** Estimated reductions in cardiovascular risk for those remaining on treatment.

Treatment	Risk factor reduction	Proportional risk reduction*						No. needed to treat for 5 yrs to prevent 1 major event	
		CHD	Ischaemic stroke	Haem-orrhagic stroke¶	Major extra-cranial bleed	Any major event -moderate risk pop^n^ §	Any major event - high risk pop^n^ §	Moderate risk pop^n^ §	High risk pop^n^ §
Blood pressure lowering^16^	10 mmHg lower SBP	22%	41%	41%	0%	26%	29%	40	9
Cholesterol lowering^5^	0.8 mmol/L lower LDL	35%	23%	0%	0%	26%	27%	40	9
Aspirin^14^	Not applicable	20%	17%	−39%	−54%	8%	13%	125	20
All three treatments		60%	62%	18%	−54%	46%	53%	18	4

*See Methods. Proportional effects from systematic reviews[5], [17], [20] and given by (1-RR)*100%, where RR is relative risk. Proportional effects of BP and cholesterol lowering emerge fully after about a year and may vary slightly by age; those for 60–69 year group shown here.

¶ Proportional effects of blood pressure lowering on haemorrhagic stroke and ischaemic stroke assumed to be the same as for total stroke, as most trials have not reported on stroke subtypes. No effect of statins on haemorrhagic stroke is assumed, reflecting the overall results from statin trials.^8^

§ Any major event  =  CHD, ischaemic stroke, haemorrhagic stroke or major extra-cranial bleed. Assumes pre-treatment annual rates of CHD, ischaemic stroke, haemorrhagic stroke and major extra-cranial bleed of 1.0%, 0.6%, 0.1%, and 0.2% (ie. moderate risk - the average for this trial population[13], [20], [51], [52]) and of 4.0%, 3.0%, 0.3% and 0.4% (high risk - expected for people with symptomatic coronary artery disease[29], [53]). These event rates will vary according to many factors, especially age and disease history.

Footnote: Trials indicate this formulation would also affect other vascular and related outcomes, but in most patient populations these would have less clinical impact due to lower incidence and/or severity. Blood pressure lowering would reduce heart failure incidence (by about a quarter), headache and renal events;[17], [54], [55], [56]aspirin would reduce venous thromboembolism.[57;, 1994 #1665] An approximately neutral overall effect on diabetes incidence is expected: ACE-inhibitors reduce risk[58] but this would be offset by small increases in risk conferred by the low-dose thiazide[59] and statin.[60] Effects on major non-vascular events would also occur, but similarly the absolute effects would mostly be small: the thiazide would reduce renal calculus and fracture, and increase gout;[17], [54] the statin will cause rhabdomyolysis (in less than 1 per 10,000 patient years[61]) and long-term aspirin can be expected to reduce gastrointestinal cancer by about one-third and all solid cancers by about one-fifth.[62]

## Discussion

These results show that treatment with this polypill achieved sizeable reductions in SBP and LDL-cholesterol. These risk factor reductions, together with the findings of systematic reviews of the component medicines, indicate that this treatment can be expected to more than halve cardiovascular risk. Starting treatment with this polypill caused side effects sufficient to stop treatment in about 1 in 20 people. Other less serious side effects occurred in about 1 in 8 people, with most becoming apparent after just a few weeks of treatment.

There are several limitations of this study. The relatively short follow-up precluded assessment of the long-term rates of drop-out. It is well recognized, for example, that gastric bleeding due to aspirin can occur months or even years after starting treatment. However, placebo-controlled trials of the separate components of this polypill show that most long-term drop-out is not related to side effects (i.e. drop-out rates in the placebo group are much more than half those in the active group) and that long-term dropout rates are much lower than those observed early after starting treatment. Nonetheless, the effects on cardiovascular events estimated here only apply to those staying on treatment long-term. While characteristic side effects were the only ones evident, the design precluded definitive attribution of which component caused which side effects. The patient population represented a relatively narrow group, having raised cardiovascular risk and no existing indications for any of the medicines. However, history of symptomatic cardiovascular disease does not modify the extent of risk factor reductions, which are likely to be broadly generalisable.[4], [5] Finally, the predicted reductions in cardiovascular risk are based on reductions in risk factor levels and, while it appears that blood pressure or LDL-cholesterol reductions account for most or all of the benefits,[17], [18], [19], [20], [21], [31], [32] these are nonetheless indirect estimates.

Two previous trials have assessed the effects of polypill treatment compared to control on risk factor reductions, tolerability and estimated cardiovascular risk, and their results are compared with the current study and Wald and Law's original predictions in Table 4. The Indian Polycap Study (TIPS) randomized 2,053 individuals without cardiovascular disease, aged 45–80 years and with one or more risk factors, to 12 weeks treatment with the Polycap (hydrochlorothiazide 12·5 mg, atenolol 50 mg, ramipril 5 mg, simvastatin 20 mg and aspirin 100 mg), or to one of eight other groups: aspirin alone, simvastatin alone, hydrochlorothiazide alone, three combinations of the two blood pressure lowering drugs, three blood pressure lowering drugs alone, or three blood pressure lowering drugs plus aspirin.^[23]^ This design allowed demonstration that the risk factor reductions from each treatment modality were essentially the same in the presence and absence of other treatments.^[23]^ Malekzadeh et al conducted a double-blind randomised placebo controlled trial in residents of Golestan, Iran.[33] Following an 8-week placebo run-in period, 475 participants, aged 50 to 79 years, without cardiovascular disease, hypertension or hyperlipidaemia were randomised to fixed-dose combination therapy (aspirin 81 mg, enalapril 2.5 mg, atorvastatin 20 mg and hydrochlorothiazide 12.5 mg) or placebo for a period of 12 months. Both trials had relatively high non-attendance at final follow-up: 16% in TIPS and 27% in the Malekzadeh et al trial (22% in the control group and 33% in the polypill group, p = 0.02 for difference), compared to 1% in the current trial. Therefore TIPS and in particular the Malekzadeh et al trial were more prone to bias, especially when assessing side effects since these are often associated with loss to follow-up.

**Table 4:** Comparison with previous polypill studies.

	Formulation	Blood pressure (mmHg)		LDL-cholesterol (mmol/l)		Placebo-corrected absolute excess of side effects**		Estimated proportional risk reduction	
		Baseline level	Reduction	Baseline level	Reduction	Sufficient to stop treatment in short term	Causing any symptoms	CHD	Stroke
Wald and Law* [4], [5], [6]	Statin (eg. simvastatin 40 mg), three ½ strength blood pressure drugs, aspirin 75 mg	150/90	20/11	4.8	1.8	2%	8–15%	86%	74%
TIPS[23]	Simvastatin 20 mg, hydrochlorothiazide 12·5 mg, atenolol 50 mg, ramipril 5 mg, aspirin 100 mg	134/85	7/6	3.0	0.7	n/a	n/a	62%	48%
Malekzadeh et al [33]	Atorvastatin 20 mg, enalapril 2.5 mg, hydrochlorothiazide 12.5 mg, aspirin 81 mg	128/79	5/2	3.0	0.5	n/a	n/a	34%	21%
Current trial	Simvastatin 20 mg, hydrochlorothiazide 12·5 mg, lisinopril 10 mg, aspirin 75 mg	134/81	10/5	3.7	0.8	5%	16%	60%	56%

*Estimated rather than observed risk factor reductions and side effects. Predictions for formulation without folic acid.

**Not estimable for TIPS due to lack of placebo control and side effects not reported reliably in Malekzadeh et al trial (see Discussion). Side effects ‘causing any symptoms’ refers to those observed in 12 weeks treatment for current trial and predictions for both short and long term treatment by Wald and Law. This excess was estimated at 8% for a formulation containing a thiazide, angiotensin II receptor blocker and calcium channel blocker and 15% for a formulation containing a thiazide, beta-blocker, and ACE inhibitor.

The risk factor reductions in TIPS were comparable in size to those observed in the current trial, even though the Polycap contained an additional blood pressure lowering agent. At the end of 12 weeks, 66/412 (16%) people in the Polycap group had stopped taking study treatment compared to 34/189 (18%) in the current trial. TIPS could not estimate total excess (ie. placebo-corrected) side effects, since each comparison group contained at least one active component. However it did report no clear difference in side effect rates between the different active groups. The risk factor reductions in the Malekzadeh trial were notably lower than both TIPS and the current trial, but this may well be due to low baseline levels (which were differential between the groups for blood pressure, p<0.0001), loss to follow-up and non-adherence. The reported rates of side effects were also very low, with only 40/475 (8%) participants reporting reluctance to take study treatment and only 2/475 (0.4%) reporting adverse drug reactions. This is likely to be in large part attributable to the combined impact of loss to follow-up, under-reporting and non-adherence.

One further active-controlled trial has recently been completed, in which 216 individuals from Sri Lanka who were aged over 50 years old if female and over 40 years if male, and who had an estimated 10-year total CVD risk score >20%, based on WHO CVD risk prediction charts, were randomized to a polypill (containing 75 mg aspirin, 20 mg simvastatin, 10 mg lisinopril and 12.5 mg hydrochlorothiazide) or to standard practice.[34] The results suggested similar reduction in risk factors and predicted cardiovascular risk in both groups.

This is the first trial to empirically test Wald and Law's predictions of side effects attributable to a polypill: Table 4 shows that the observed excess of side effects is considerably greater than that predicted. The risk factor reductions seen in the current trial are also about half the size predicted by Wald and Law,[6] mostly because those estimates were based on higher baseline risk factor levels, use of a more potent statin and an extra blood pressure lowering agent. Interestingly however the estimated reductions in CHD and stroke from this polypill are only about 25–30% smaller than those of Wald and Law. This is because of the diminishing marginal returns from additional reductions in single risk factors, and the multiplicative benefits of adding different treatment modalities ie. less risk reduction from one modality leaves ‘more to slice off’ for the next modality.

What are the implications of these findings for research and clinical practice? Among patients at low-to-moderate global cardiovascular risk, further work is required on polypill formulations and target patient populations. This trial suggests that the short-term tolerability of a polypill is not as good as previous predictions or trials have suggested, although still nonetheless causing no symptoms in 5 out of 6 people treated. Most side effects, including virtually all major ones, would be due to aspirin, and the inclusion of aspirin in combination treatment provides modest net benefits,[20] although recent data showing that aspirin reduces the incidence of cancer will change this risk-balance equation back again. Nonetheless, even among patients at moderately elevated risk, such as the average in this trial (which is considerably higher than the risk faced by individuals with, for example, uncomplicated hypertension, dyslipidaemia or diabetes) the absolute benefits of aspirin would be small. For such individuals whose risk has first been reduced by blood pressure and cholesterol lowering, aspirin would only avoid a major event in every thousand or more patients per year. Polypills based on blood pressure and cholesterol lowering agents are therefore required, along with research on their benefits and risks compared to usual care. An area of controversy will be whether such trials should assess hard cardiovascular endpoints (taking many years to complete) or just measure side effects and BP and LDL reduction, given the conclusive evidence of the event reduction from individual treatments, the lack of interaction between the treatments, and the finding that most or all of the benefits are due to the extent of BP or LDL reduction. [17], [19], [20], [22] One further issue is that although efficacy of blood pressure and cholesterol lowering has been clearly established well below historical ‘hypertension’ and ‘dyslipidaemia’ thresholds,[21], [35], [36] indications currently approved by regulatory authorities and much clinical practice is restricted to those with ‘hypertension’ or ‘dyslipidaemia’ (regardless of the level of cardiovascular risk).

Among patients with a history of occlusive vascular disease, further evidence on efficacy for individual medication classes is not required, since this has been established with clinical trials involving many tens of thousands of patient over half a century. All major cross-disciplinary guidelines[9], [23], [37] recommend some form of blood pressure lowering, cholesterol lowering and antiplatelet therapy in patients with vascular disease. Research is therefore only required on the comparative roles of polypill-based treatment compared to usual care in delivering these therapies long-term. People with previous symptomatic vascular disease are relatively easily identified, more motivated to take treatment and account for almost half of all major cardiovascular events. Among this group the benefits of this combination therapy substantially outweigh the side effects, and one could reasonably expect similar-sized benefits in asymptomatic patients at equivalently high risk, who comprise about 5% of the adult population.[38] Yet the vast majority of highest-risk people globally do not receive such treatment long-term. In economically developed countries most are now prescribed recommended medicines after an acute event, but many do not continue: only one- to two-thirds of people with a history of vascular disease take antiplatelet, blood pressure lowering and statin therapy long-term.[39], [40], [41], [42] In economically developing countries, where 80% of the global burden of cardiovascular disease occurs,[43] very few receive these medicines in the short or long-term.[44], [45] With current approaches, these treatment gaps are closing very slowly.[46] Increasing access to treatment is a potentially highly cost-effective strategy[47], [48], [49] and alone could achieve most of WHO's goals for reducing non-communicable disease.[50]

## Supporting Information

Protocol S1 Trial Protocol
(DOC)

Checklist S1 CONSORT Checklist
(PDF)
