# Correction: An international randomised placebo-controlled trial of a four-component combination pill (“polypill”) in people with raised cardiovascular risk

**DOI:** 10.1371/journal.pone.0225924

**Published:** 2019-11-25

**Authors:** The PLOS ONE Staff

The Supporting Information files, S1 Protocol and S1 Checklist, cannot be downloaded in the original article. Please view the files below.

## Supporting information

S1 Checklist CONSORT checklist. (DOC)

S1 Protocol Trial protocol. (PDF)
