# Supplementary material for: An International Randomised Placebo-Controlled Trial of a Four-Component Combination Pill (“Polypill”) in People with Raised Cardiovascular Risk
Source: PLoS One. 2011 May 25;6(5):e19857. doi: 10.1371/journal.pone.0019857 (PMC3102053; doi:10.1371/journal.pone.0019857)
Supplement: Protocol S1 — Trial Protocol (DOC) [file pone.0019857.s001.doc]

PILL Pilot

Programme to Improve Life and Longevity

A randomised placebo-controlled trial of fixed-dose combination medication in people at raised risk of cardiovascular disease (ACTRN012607000099426)

TRIAL PROTOCOL

Version 7.0

29 June 2009

CONTACT DETAILS:
International Coordinating Centre
Clinical Trials Research Unit
Department of Medicine, The University of Auckland
Private Bag 92019, Auckland, New Zealand

Tel: (+64 9) 373 7999. Fax: (+64 9) 373 1710
Email: pill@ctru.auckland.ac.nz

Courier address:
School of Population Health, Tamaki Campus
Cnr Merton & Morrin Roads, Glen Innes
Auckland, New Zealand

Confidential. Not for public circulation or reproduction.

Principal investigator
Professor Anthony Rodgers
Email: arodgers@george.org.au

Project manager
Angela Wadham
Email: a.wadham@ctru.auckland.ac.nz

Project Funders

International:	Wellcome Trust
New Zealand:	Health Research Council of New Zealand
	National Heart Foundation of New Zealand
Australia	National Health & Medical Research Council
United Kingdom:	British Heart Foundation

Polypill provided by:

Dr Reddy's Laboratories Limited. 7-1-27 Ameerpet, Hyderabad 500 016, Andra Pradesh, India. Ph: (+91) 40 2373 1946. Fax: (+91) 40 2375 0984.

Protocol Version History

Version Number	Date
Version 1	6 October 2006
Version 2.0	2 April 2007
Version 3.0	12 October 2007
Version 4.0	25 February 2008
Version 5.0	8 May 2008
Version 6.0 (Submitted only in The Netherlands)	8 April 2009
Version 7.0	29 June 2009

Principal Investigator
Professor Anthony Rodgers

Document Approval Signature: A Rodgers

Date: 29 June 2009

Email: arodgers@george.org.au

TABLE OF CONTENTS

1. OVERVIEW 6
1.1 Basic trial plan schematic 7

2. BACKGROUND 8
2.1 Trial intervention 10
2.2 Trial rationale 10

3. AIMS 10

4. TRIAL DESIGN 11
4.1 Inclusion/exclusion criteria 11

5. RECRUITMENT 12
5.1 Self-referral to a dedicated trial centre 12
5.2 Identification and invitation in primary and/or secondary care and the community 13

6. ETHICS AND REGULATORY APPROVAL 13
6.1 Ethics committees 13
6.2 Regulatory committees 13
6.3 Informed consent 13

7. BLINDING 13

8. RANDOMISATION 13

9. TRIAL TREATMENT AND ONGOING MANAGEMENT 14
9.1 Trial treatment 14
9.2 Concomitant treatment 14
9.3 Premature discontinuation of trial treatment 15
9.4 Unblinding of trial treatment 16

10. TRIAL OUTCOMES 16

11. DATA COLLECTION AND FOLLOW-UP 17
11.1 Registration 18
11.2 Baseline visit 18
11.3 Week 2 and 6 follow-up visits 19
11.4 End of trial visit 19
11.5 Post trial assessment 20
11.6 End of trial 20

12. ADVERSE EVENT REPORTING 20
12.1 Adverse events 20
12.2 Reporting of serious adverse events and serious suspected adverse drug reactions 22
12.3 Adverse event reporting flow chart 23

13. MONITORING 24
13.1 Close-out visit 24
13.2 Audit 24

14. TRIAL TREATMENT 25
14.1 Manufacture 25

14.2 Identification 25
14.3 Packaging and labelling 25
14.4 Handling and dispensing 25
14.5 Trial treatment supply records at Regional Coordinating Centres 25
14.6 Trial completion or termination 26

15. TRIAL POWER AND PLANNED ANALYSES 26

15.1 Sample size 26
15.2 Statistical analysis 26

16. ADMINISTRATIVE SECTION 28

16.1 Trial registration 28
16.2 Roles and responsibilities 28
16.3 Training 30
16.4 Adherence to the protocol 31
16.5 Protocol revisions 31
16.6 Case report form procedures 32
16.7 Data confidentiality and security 32
16.8 Periodic reports 32
16.9 Records retention 32
16.10 Insurance 32
16.11 Ownership of data and publication 33
16.12 Funding 33

REFERENCES 34

APPENDIX 1 –– INFORMED CONSENT PROCEDURES 35

APPENDIX 2 – ICH GCP PRINCIPLES 36

APPENDIX 3 – PROTOCOL SIGNATURE PAGE 37

1. Overview

A randomised placebo-controlled trial of fixed-dose combination medication
in people at raised risk of cardiovascular disease.

Background and rationale. One of the most hotly debated issues in cardiovascular disease (CVD) research is whether a "polypill" (a new combination cardiovascular medication containing aspirin and agents to lower blood pressure and cholesterol) can really reduce CVD by three-quarters or more. To assess this reliably will require a long term clinical trial of many thousands of participants. This protocol is for a pilot trial that will assess the efficacy and tolerability of the polypill.

Trial design. Randomised, placebo-controlled, parallel-group trial (n=400) of the polypill in individuals at raised risk of cardiovascular disease.

Aims. To measure the efficacy (change in systolic blood pressure and LDL cholesterol) and tolerability of a polypill in individuals with raised risk of a cardiovascular event. Secondary aims are to measure adherence, the change in diastolic blood pressure, total cholesterol, HDL cholesterol, total cholesterol:HDL cholesterol ratio, non HDL cholesterol and triglycerides, frequency of switching to open-label treatment, change in estimated CVD risk, serious adverse events and all adverse events.

Patient recruitment. Participants will include those who have an absolute risk of a major cardiovascular event in the next 5 years of 7.5% or more (based on a Framingham risk function and presence of other major cardiovascular risk factors) but who do not meet current guidelines for treatment with aspirin, or blood pressure lowering or cholesterol lowering medications. To be eligible, there must be no clear indication for, or contraindication to, the polypill components. Participants will be recruited using primary and/or secondary care screening, referrals and media advertisements. It is proposed that 400 participants will be recruited in total – between 20 -175 participants from each of Australia, Brazil, India, the Netherlands, New Zealand, the United Kingdom and the United States.

Randomisation and trial treatment. Eligible individuals willing to participate in the trial will be randomised to 12 weeks treatment with the polypill or to an identical matching placebo:

- *Polypill*: aspirin 75mg, simvastatin 20mg, lisinopril 10mg, hydrochlorothiazide 12.5mg.
- *Placebo*: identical matching placebo.

The polypill and placebo will be provided by the investigator at the Local Trial Centre. Both groups will also receive information about smoking cessation (if applicable) and how to follow a healthy heart diet. They will be advised to increase physical activity and lose weight if needed.

Other treatments will be at the discretion of the responsible physician.

Data collection and follow-up. Participants will be followed-up for 12 weeks during the treatment period. Blood pressure will be measured at baseline, weeks 2, 6 and 12. Fasting lipids will be measured at baseline and week 12. Tolerability will be assessed at weeks 2, 6 and 12, as will trial treatment adherence and adverse events. Adverse event data will also be collected 4 weeks after the completion of the treatment period.

Outcomes. *Primary outcomes:* Efficacy (systolic blood pressure, LDL cholesterol) and tolerability (the proportion who withdraw from trial treatment). *Secondary outcomes:* Treatment adherence (measured by pill count), diastolic blood pressure, total cholesterol, HDL cholesterol, total cholesterol:HDL cholesterol ratio, non HDL cholesterol, triglycerides,

frequency of switching to open-label treatment, estimated CVD risk, serious adverse events and all adverse events.

1.1 Basic trial plan schematic

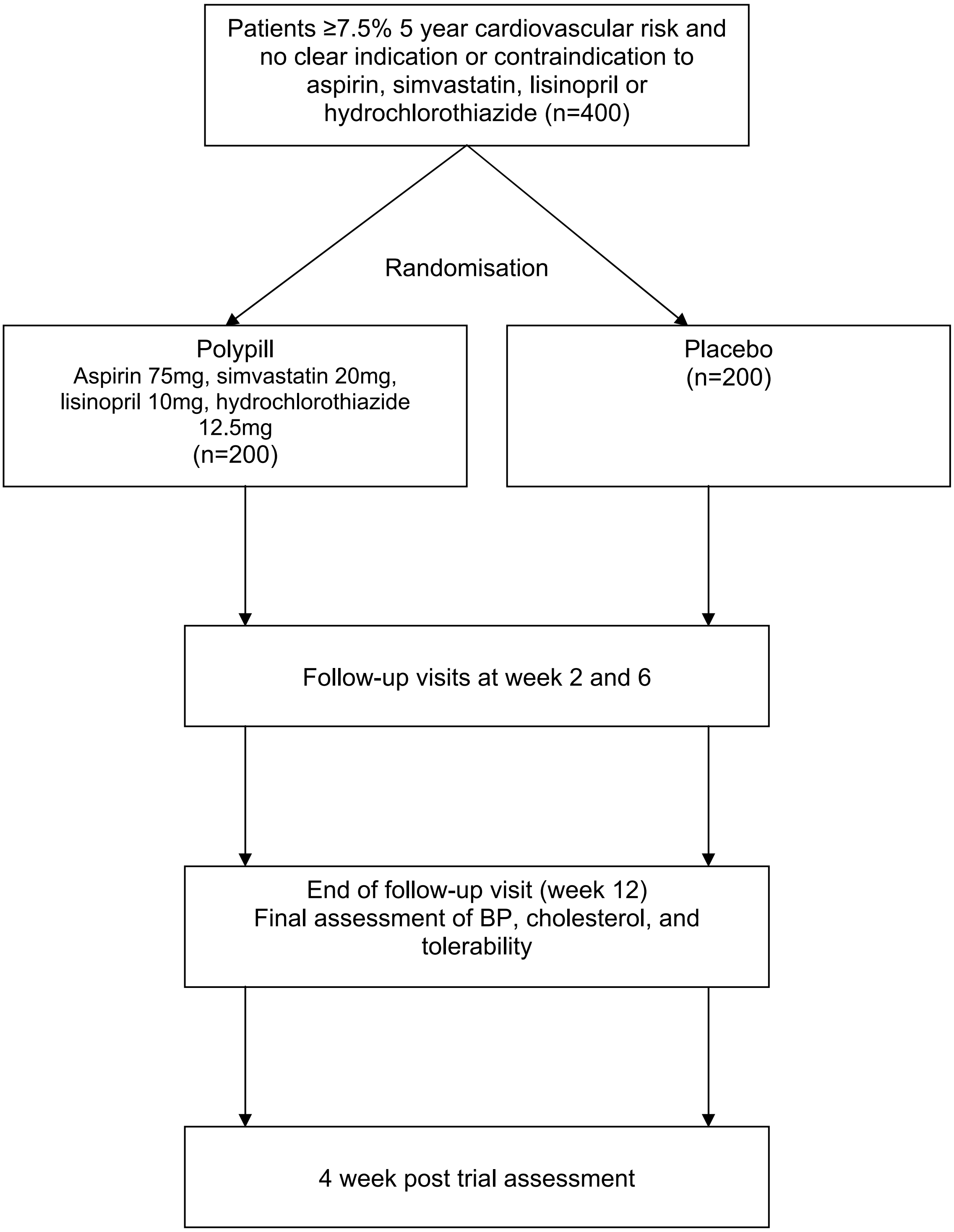

2. Background

Globally, high blood pressure and cholesterol cause more than half of all cardiovascular disease.[1] Both are leading risks to health in economically developed countries and are increasingly important in developing countries.

Figure 1– Joint effects of blood pressure, cholesterol and smoking on coronary risk in MRFIT cohort

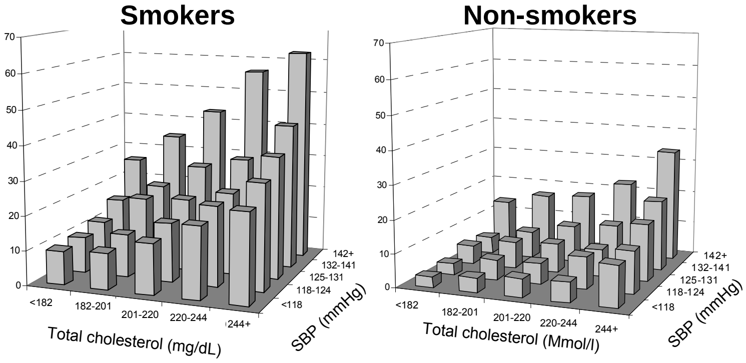

The increased risk of a cardiovascular event is not restricted to those with "hypertension" or "hyper-cholesterolaemia", but is continuous down to a blood pressure of at least 115/75 mmHg and total cholesterol level of 4.0 mmol/L (135 mg/dl). There is now clear evidence from large randomised trials in people with established cardiovascular disease of benefits of blood pressure and cholesterol lowering and antiplatelet therapy, largely irrespective of baseline risk factor levels.[2 3] Data from the MRFIT study[4] show (Figure 1) that the relationship between cholesterol and coronary disease is strong and graded across all levels of blood pressure, that the relationship between blood pressure and coronary disease is strong and graded across all levels of cholesterol, and that tobacco smoking amplifies these risks across the board. Overall, the epidemiological evidence from large prospective cohorts clearly indicates the potential benefits of lowering blood pressure and cholesterol in those with average or even below average levels (which are currently termed "normal levels").[2] Recently, clinical trials have confirmed the benefits of blood pressure and cholesterol lowering in high-risk patients who do not have "hypertension" or "hyper-cholesterolemia" (Figures 2 and 3).[5 6]

There is evidence that the burden of CVD among those without CVD but at elevated risk is substantial. Using MRFIT CVD risk scores for a cross-section of the Australian population, the 30% of the CVD-free Australian population most at risk from CVD was predicted to account for over 50% of the population's primary CVD.[7]

Figure 2 - Reduction of stroke with combination blood pressure lowering therapy in PROGRESS, by baseline BP

Blood pressure (mmHg)	Stroke events active	Stroke events placebo	Favours active / Favours placebo	Risk reduction (95%CI)
SBP >160	57	106		47% (27-62%)
SBP 140-159	54	87		41% (16-58%)
SBP <140	39	62		39% (19-59%)
DBP >95	27	68		62% (41-76%)
DBP 85-94	65	99		34% (22-53%)
DBP <85	58	88		37% (22-55%)
**Total**	**150**	**255**		**43% (30-54%)**

Hazard Ratio: 0.4 1.0 2.0

Figure 3: Reduction in cardiovascular events with cholesterol lowering in Heart Protection Study, by baseline cholesterol

Baseline cholesterol (mg/Dl)	Events active	Events placebo	Favours active / Favours placebo	Risk ratio* (95%CI)
TC >232	929	1149		0.81 (0.75-0.87)
TC 193-232	744	964		0.77 (0.71-0.84)
TC<193	360	472		0.77 (0.68-0.87)
LDL >135	951	1183		0.81 (0.75-0.87)
LDL 116-135	484	646		0.74 (0.67-0.82)
LDL <116	598	756		0.80 (0.72-0.88)
**Total**	**2033**	**2585**		**0.79 (0.75-0.83)**

Risk Ratio: 0.6 0.8 1.0 1.2 1.4

Very few individuals receive combination therapy for primary prevention, even though many with multiple, moderate elevations of risk factors are at raised cardiovascular risk. We have recently completed analyses of data from the Royal New Zealand College of General Practitioners database which demonstrate these “treatment gaps”.[8] These are particularly marked for people in the moderate risk range (Table 1).

Table 1 Prescription of CV medication according to CV history and absolute risk among NZ men aged 45 years+ and NZ women aged 55 years+

	**CV history**	**No CV history**					
**5-yr CV risk (number of individuals)**	**≥20% (3 855)**	**≥20% (570)**	**≥15-<20% (515)**	**≥10-<15% (1005)**	**≥5-<10% (1507)**	**<5% (979)**	**Risk not estimable (16 950)**
BP lowering medication	67%	62%	58%	53%	44%	30%	23%
Lipid lowering medication	33%	21%	23%	20%	21%	15%	2%
Combination (BP & lipid lowering)	*28%*	*16%*	*16%*	*14%*	*14%*	*8%*	*1%*

The New Zealand results are very similar to US data from the National Health and Nutrition Examination Survey, NHANES, (Figure 4) which showed about half of individuals at >7.5% risk do not currently receive medicines for blood pressure or cholesterol lowering. This is likely to be because they have neither suffered a cardiovascular event nor exhibited an individual risk factor at a level above the currently recommended threshold for treatment.

Figure 4 – Estimated numbers of people in the US receiving blood pressure and cholesterol lowering treatments, by absolute cardiovascular risk (NHANES 1999-2002, aged 40+ yrs, N=5,636)

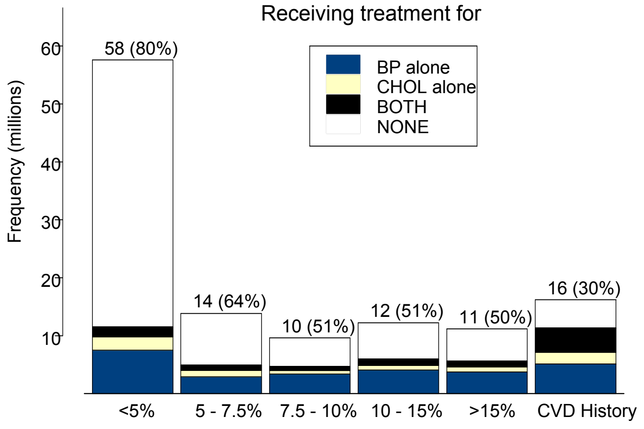

Estimated 5yr CVD absolute risk (Framingham function)
Numbers above each bar are number of people within each risk category (percentages not taking blood pressure or cholesterol lowering medications)

Cardiovascular disease is a major cause of health inequalities because there are major inequities in access to and use of preventive strategies. Ethnic minorities and indigenous peoples often have poor access to primary care and a lower pharmaceutical expenditure per capita than among the general population despite greater rates of cardiovascular disease.[9] Many countries have restrictions on the availability of statins due to their initial cost, and these barriers further disadvantage lower socio-economic groups.

2.1 Trial intervention

A polypill has been manufactured to Good Manufacturing Practice standards by Dr Reddy's Laboratories Limited, with bioequivalence to the separate components. Details regarding preclinical and clinical development are found in the Investigator's Brochure.

The polypill contains aspirin 75mg, simvastatin 20mg, lisinopril 10mg and hydrochlorothiazide 12.5mg.

The polypill dose regimen is one tablet in the evening with food. The dose of aspirin was chosen on the basis of evidence indicating that lower doses of aspirin (50-125 mg/day) achieve similar benefits in terms of reduction in coronary and stroke events, and lower risks of serious side effects, compared with higher doses (160-1500 mg/day).[10] Two blood pressure-lowering agents at less than full dose are included to maximise blood pressure reduction (as greater risk reductions are produced with more intensive blood pressure lowering), while reducing the risks of side effects from any one drug. [3 11 12] Lisinopril was used in the ALLHAT trial; 10 mg per day is the usual starting dose.[13] A low-dose thiazide was included due to its recommended first line use in hypertension and low side effect profile.[13]

An extensive body of knowledge exists about the individual components of the polypill. These medicines are commonly taken concurrently and unexpected adverse reactions are considered highly unlikely.

2.2 Trial rationale

The use of fixed-dose combination therapy to prevent cardiovascular disease has generated enormous debate since Wald and Law advocated the widespread use of a "polypill" for the prevention of cardiovascular events, including in the context of primary prevention.[14] The net benefits of combined treatment with aspirin, blood pressure lowering and cholesterol lowering agents may be substantial, since each intervention works in addition to the others. Based on extrapolation from previous trials, a more than halving of cardiovascular risk might be expected (increasing to a two-thirds or greater reduction after the first few years, as the full effects of the component medications accrue).[14] The cost of such treatment containing off-patent medications is very low. Side effects could be minimised by using low-dose combinations of blood pressure lowering agents and low-dose aspirin. Based on extrapolation from previous trials, side effects would be outweighed several-fold by the net benefits among individuals at moderate-to-high cardiovascular risk.[14] However, direct evidence from randomised trials of both joint benefits and side effects is lacking. For these reasons, a large randomised endpoint trial is urgently required. Hence the goal of this research is to conduct a randomised trial to provide preparatory data for a large clinical endpoint trial of the polypill in people at raised cardiovascular risk. This pilot trial will be undertaken by an international research network in Australia, Brazil, India, New Zealand, the US and the UK. The pilot will provide essential information for demonstrating the feasibility and appropriate power of the main trial.

3. Aims

The primary aim of the trial is to evaluate whether a polypill (containing low dose aspirin, a statin and two blood pressure lowering medicines) results in improved systolic blood pressure and LDL cholesterol levels and is tolerable compared with placebo in individuals at raised risk of cardiovascular disease. Secondary aims are to measure adherence, change in diastolic blood pressure, total cholesterol, HDL cholesterol, total cholesterol:HDL cholesterol ratio, non HDL cholesterol, and triglycerides, frequency of switching to open-label treatment, estimated CVD risk, serious adverse events and all adverse events.

4. Trial design

The trial is an international, randomised, double-blind, placebo controlled, parallel-group design trial (n =400).

4.1 Inclusion/exclusion criteria

The trial targets individuals who have an absolute risk of a major cardiovascular event in next 5 years of at least 7.5% but who do not meet current guidelines for treatment with aspirin, or blood pressure-lowering or cholesterol-lowering medications.

Individuals are eligible for inclusion if all of the following criteria are satisfied:

- Adults ($\geq$ 18 years) with a cardiovascular disease (CVD) risk over 5 years of at least 7.5%, determined by the Framingham risk function (Anderson, 1991) using data on age, gender, blood pressure, total cholesterol, HDL cholesterol, diabetes status and cigarette smoking status. Left ventricular hypertrophy will be assumed to be absent for the purpose of CVD risk calculation. Those with an estimated 5 year risk of 5.0 – < 7.5 % risk would also be eligible with two or more additional risk factors of:
    - Body mass index (BMI) > 30 kg/m^2, waist circumference > 102 cm in men or > 88 cm in women
    - Heart rate > 80 beats/min
    - Fasting glucose 5.6 - <7 mmol/L
    - Triglycerides > 1.7 mmol/L
    - Family history of premature coronary heart disease or ischaemic stroke in a first degree male relative before the age of 55 years or a first degree female relative before the age of 65 years
    - Glomerular filtration rate (GFR) < 60 mL/min

- No indication or contraindication for treatment with low-dose aspirin, angiotensin-converting enzyme (ACE) inhibitor, low-dose diuretic or statin.

- The participant is able to give informed consent.

Individuals will NOT be eligible if there is one or more of the following:

- Clear indication for antiplatelet or blood pressure lowering or cholesterol lowering medications. This includes: current treatment with blood pressure or cholesterol lowering medicines, existing CVD, or individuals with LDL cholesterol, systolic blood pressure or estimated CVD risk values above those recommended for treatment by local guidelines.

- Diabetes mellitus.

- GFR $\leq$30 ml/min/1.73m^2

- Contraindication to any of the components of the polypill (aspirin, ACE inhibitor, diuretic or statin). This includes: ALT > 3 times the upper limit of normal, history of cirrhosis or hepatitis, suspected renal artery stenosis, recent gastrointestinal bleeding (within the last year), pregnant, breastfeeding or a female of child-bearing potential not on reliable contraception and also any circumstance where ongoing medication might lead to potential adverse drug interaction with components of the polypill (see section 9.2 Concomitant treatment for list of significant drug interactions). The full list of contraindications from the Investigator's Brochure is:

    - Hypersensitivity to aspirin, lisinopril, hydrochlorothiazide, or simvastatin.
    - Hypersensitivity to any of the excipients of the tablet.
    - Hypersensitivity to NSAIDs.
    - Hypersensitivity to sulphonamide drugs.
    - Children and teenagers with chickenpox or influenza symptoms (risk of Reye's syndrome from aspirin).
    - Syndrome of asthma, rhinitis, and nasal polyps. Bronchospasm or urticarial reactions to aspirin.
    - ACE inhibitor–induced angioedema, history of angioedema; hereditary or idiopathic angioedema
    - Pregnancy or lactation. Recent history (3 months) of pregnancy.
    - Cardiogenic shock, or overt cardiac failure.
    - Severe, uncorrected electrolyte disturbances of Na+, K+, Mg++, or Ca++.
    - Active peptic ulcer, or other active bleeding.
    - Active gout.
    - Active liver disease. Unexplained persistent elevations in hepatic transaminases.
    - Myopathy secondary to other lipid lowering agents.
    - Anuria / oliguria, or uraemia.

- Any reason, medical condition, ongoing medication or significant disability that would prevent the participant complying with trial consent, treatment and follow-up procedures or potentially jeopardise her / his medical care.

5. Recruitment

It is proposed that 400 participants will be recruited in total – between 50 - 100 participants from each of Australia (Sydney), Brazil (Porto Alegre / Sao Paulo), India (Hyderabad), the Netherlands (Utrecht), New Zealand (Auckland), the UK (London) and the US (Minneapolis).

Two main models of recruitment are envisaged, with individual countries choosing their preferred method(s). The number of potential participants screened and participants recruited will be recorded from each method.

5.1 Self-referral to a dedicated trial centre

Any recruitment campaigns using advertising media should contain clear references to the experimental nature of the trial and should not be inappropriately promotional.

Interest is generated from television and radio advertising, talk shows, health issues programmes, advertising in newspapers and websites and mass mailings. Interested persons are directed to the internet where they complete a screening questionnaire. On completion of the questionnaire, the participant’s estimated 5 year cardiovascular disease risk level is calculated using the Framingham algorithm, with imputed values from national age- and sex-specific averages for blood pressure and cholesterol. Those with an estimated risk between 5 and 20% will be identified as potentially eligible and invited to self-refer to the Local Trial Centre where their risk level will be more accurately measured to confirm eligibility. Some regions may put in place an IVRS (Interactive Voice Response System) so potential participants can have their eligibility screened over the phone.

This method was tested in a New Zealand Health Research Council funded recruitment pilot and found to be resource efficient. Over a period of 7 weeks 235 questionnaires were completed - 50% IVRS, 50% website. This was likely to be a conservative estimate of the potential of media recruitment given most advertising was done over the Christmas period. From the completed questionnaires, 48 participants (21%) were identified as potentially eligible. Of these, 24 participants attended a clinic and 11 (5% of the total completed

questionnaires) were found to be eligible for the trial with an estimated cardiovascular disease risk level between 7.5 and 15%.

5.2 Identification and invitation in primary and/or secondary care and the community

This may involve active screening as well as receiving referrals from primary and/or secondary care practitioners, members of the public and community health groups. Screening will be performed by the investigator in clinics willing to participate in the trial using cardiovascular risk electronic decision support software (e.g. PREDICT™) or searching electronic clinical records. The investigator will then review the electronic notes of patients identified by the queries for eligibility - reviewing diagnoses, medications and the information required for cardiovascular risk calculation (age, gender, systolic blood pressure, cholesterol, smoking status and diabetes status). All patient information and an electronic copy of the queries will be stored in the clinic for the duration of the trial. In some populations the workplace may be an important source of recruitment.

6. Ethics and regulatory approval

6.1 Ethics committees

Ethical approval of the trial protocol and protocol-related documents will be sought from the appropriate local and/or national ethics committees by each Regional Coordinating Centre.

6.2 Regulatory committees

The polypill is an unapproved medicine. Therefore approval to use the polypill in a clinical trial will be sought from appropriate national regulatory authorities before the start of the trial in each participating country.

6.3 Informed consent

Written informed consent will be obtained by the investigator (Appendix 1).

7. Blinding

Participants and investigators will be blinded to trial treatment allocation. During the review of the results within the trial team (after trial completion), all investigators will be blinded to treatment allocation (all results will be presented as treatment A and B). The results will be unblinded once the final statistical report has been completed.

8. Randomisation

Eligible individuals will be randomised to trial treatments using a central, computer-based randomisation service accessible by internet. The randomisation service will be provided by the International Coordinating Centre. A minimisation algorithm will include age, sex and centre. Individuals will be randomised to the polypill or matching placebo in a 1:1 ratio with a two group parallel trial design.

9. Trial treatment and ongoing management

9.1 Trial treatment

Following randomisation, participants will be provided with a 6 week supply of the polypill or an identical placebo by the investigator at a Local Trial Centre. At the week 6 visit another 6 week supply of trial treatment will be provided. The regimen is one tablet once a day, in the evening with food.

9.2 Concomitant treatment

The following significant drug interactions may occur and individuals needing long-term treatment with drugs from this list should not be included in the study:

- α_1–blockers: hydrochlorothiazide may potentiate orthostatic hypotension.
- Alendronate: worsened gastrointestinal side effects with aspirin.
- Allopurinol: lisinopril may enhance the hypersensitivity reactions to allopurinol.
- Antacids decrease the absorption of lisinopril.
- Antiplatelet agents (abciximab; anagrelide; cilostazol; clopidogrel; dipyridamole; eptifibatide; ticlopidine; tirofiban) enhanced effect (may be an advantage), and increased bleeding complications.
- Aprotinin may decrease the antihypertensive action of lisinopril.
- Azathioprine: lisinopril may enhance the neutropenic effect of azathioprine.
- β–agonists may enhance the K^+ loss by hydrochlorothiazide.
- Bezafibrate, clofibrate, fenofibrate, gemfibrozil. Substances which compete for CYP3A4 metabolism with simvastatin increase the risk of toxicity (myositis, rhabdomyolysis, hepatic).
- Bile acid sequestrants decrease the absorption of hydrochlorothiazide.
- Bosentan, rifabutin; rifampin; rifapentine, St. John's wort and phenytoin may increase the metabolism of simvastatin, decreasing its effectiveness.
- Nondihydropyridine calcium channel blockers (diltiazem; verapamil): aspirin bleeding time increased.
- Calcium supplements may cause hypercalcaemia due to reduced excretion secondary to thiazide administration.
- Cardiac glycosides (digoxin, digitoxin), dipyridamole, and disopyramide: bradycardic effect of is potentiated by hypokalaemia secondary to hydrochlorothiazide.
- Corticosteroids: aspirin may enhance gastric ulceration.
- Coumarin anticoagulants: enhanced bleeding risk.
- Cyclosporine: lisinopril may enhance the nephrotoxic effects of cyclosporine. Cyclosporine and hydrochlorothiazide reduces uric acid excretion and can lead to gout. hydrochlorothiazide enhances cyclosporine related renal toxicity.
- Cytochrome P450 CYP isoenzyme inhibitors decrease the metabolism of simvastatin, increasing the likelihood of toxicity (hepatic, myositis, rhabdomyolysis). Medicines implicated are amiodarone, nefazodone, diltiazem; verapamil, cyclosporine, grapefruit juice (>1L/day), danazol, diclofenac, imatinib, ranolazine, sildenafil, imidazole antifungals (itraconazole; ketoconazole; miconazole), macrolide antibiotics (clarithromycin; erythromycin; telithromycin; troleandomycin, but not azithromycin; dirithromycin; spiramycin), protease inhibitors (amprenavir; atazanavir; fosamprenavir; indinavir; lopinavir; nelfinavir; ritonavir; saquinavir; tipranavir), proton pump inhibitors (esomeprazole; lansoprazole; omeprazole; pantoprazole; rabeprazole).
- Dofetilide and quinine may be associated with ventricular arrhythmias which may be enhanced by hypokalaemia and hypomagnesaemia.

- Diuretics (frusemide, thiazides) may cause symptomatic hypotension with lisinopril and hydrochlorothiazide.
- Ferric gluconate (i.v.): lisinopril may enhance the hypersensitivity reactions.
- Lithium levels may be increased by lisinopril and hydrochlorothiazide.
- Non-aspirin NSAIDs may diminish the cardioprotective effect of aspirin, and the antihypertensive effect of lisinopril.
- Potassium sparing diuretics (amiloride, spironolactone, eplenerone) and potassium supplements may cause hyperkalaemia with lisinopril.
- Treprostinil increases bleeding risk with aspirin.
- Trimethoprim may cause hyperkalaemia with lisinopril.
- Valproic acid effect may be enhanced as serum concentrations increase with aspirin.
- Varicella virus containing vaccine: may cause Reye's syndrome with aspirin. Avoid aspirin for 6 weeks if possible before using varicella containing vaccines.

If one of the medications in the trial treatment becomes indicated over the course of the trial, therapy will be added as follows:

- If aspirin therapy is indicated during the trial, then open-label 75 mg aspirin can be instituted and added to the trial treatment (without the need to determine whether it was active or placebo).
- If active blood pressure lowering is needed during the trial, physicians will be advised to add open label beta-blocker, calcium channel blocker, angiotensin receptor blocker or alpha-blocker in both groups without the need to unblind. If ACE-inhibitor and/or diuretic open label treatment is needed, then open label lisinopril 10-20 mg and/or hydrochlorothiazide 12.5 mg or bendrofluazide 2.5mg could be instituted and added to the trial treatment (without the need to determine whether it was active or placebo). If higher doses or other types of ACE-inhibitor or diuretic are indicated, these would be provided as open label treatment and the trial treatment stopped.
- If cholesterol lowering is indicated during the trial, then open-label simvastatin 10-20mg could be instituted and added to the trial treatment (without the need to determine whether it was active or placebo). Open-label fibrate (with the exception of gemfibrozil) can also be added, without the need to unblind or stop the trial treatment, provided that appropriate monitoring for rhabdomyolysis is instituted. If higher doses of simvastatin or other statins or gemfibrozil are indicated, these would be provided as open label treatment and the trial treatment stopped.

All concomitant medication will be recorded on the case report forms (CRFs): generic and trade names, dose, frequency of administration.

9.3 Premature discontinuation of trial treatment

Trial treatment may be permanently or temporarily discontinued for a particular participant for one of the following reasons:

1. A definite indication for, or contraindication to, any of the active treatments becomes apparent;

2. A participant makes a personal decision to discontinue either of the randomised treatments;

3. Pregnancy (always permanent discontinuation without exception);

4. Onset of an adverse event as described in Table 3 (page 21)

5. Abnormal laboratory values as described in Table 3 (page 21)

6. Any other medical reason at the discretion of the trial doctor.

Wherever possible, randomised trial treatments should be continued throughout follow-up. If discontinuation is necessary, the possibility of recommencing therapy should always be considered. Regardless of whether the participant continues to adhere to the randomised treatment, the follow-up schedule will continue unchanged for all randomised participants.

Trial treatment should be **continued** if the participant develops an intercurrent illness that neither indicates nor contraindicates any of the individual ingredients of the polypill.

9.4 Unblinding of trial treatment

An emergency unblinding facility will be maintained for the duration of the trial by the International Coordinating Centre. The unblinding will be performed by individuals not otherwise involved in the day-to-day conduct of the trial. The facility (in English) will be available 24 hours a day, 7 days a week, with access by telephone. In general, unblinding of participants should only be performed when knowledge of the treatment allocation will influence participant management: for example after overdose of the trial treatment. Unblinding need not be performed if any of the polypill constituents have become definitely indicated or definitely contraindicated, for example:

- If treatment becomes definitely indicated, add therapy as outlined in 9.2. If trial treatment needs to be stopped and open label treatment commenced, do not unblind.
- If treatment becomes definitely contraindicated, then discontinue the trial treatment but do not unblind.

10. Trial outcomes

Primary outcomes:

- Efficacy
  - Systolic blood pressure
  - LDL-cholesterol
- Tolerability (proportion who withdraw from the trial treatment)

Secondary outcomes:

- Trial treatment adherence (pill counts)
- Diastolic blood pressure
- Total cholesterol
- HDL cholesterol
- Total cholesterol:HDL cholesterol ratio
- Non HDL cholesterol
- Triglyceride
- Frequency of switching to open-label treatment

- Estimated CVD risk
- Serious adverse events
- Adverse events
- Serum angiotensin converting enzyme (ACE) activity (subset of participants)

Blood pressure and cholesterol measures and estimated CVD risk will be assessed as change from baseline over the 12 weeks.

11. Data collection and follow-up

The procedures performed at each visit are outlined in Table 2 below. Potentially eligible participants found through screening will be invited to attend a baseline visit at the Local Trial Centre. Eligible participants will then be randomised and provided with a 6 week supply of double-blind polypill or matching placebo. They will be instructed to take one tablet daily, in the evening with food. Both groups will also receive information about smoking cessation (if applicable) and how to follow a healthy heart diet. They will be advised to increase physical activity and lose weight if needed.

Follow-up visits will be conducted by the investigator at the Local Trial Centre at 2, 6 and 12 weeks. Furthermore, the Local Trial Centre will contact participants 4 weeks after the completion of the treatment period to record the nature of any adverse events that may have occurred during this time. Participants will continue to receive non-trial related healthcare from their usual primary care practitioner. If a participant is unable to attend any of the scheduled visits then as far as possible, follow-up will be completed by other means (e.g. rescheduling appointments, making home visits, doing telephone follow-up, contacting the primary care practitioner).

Full instructions on trial measurements are given in the Manual of Procedures.

Table 2 Summary of trial visits and procedures completed at each visit

Description	Registration	Baseline visit	Follow-up visit	Follow-up visit	End of trial visit	Post trial assessment
**Timing**	**Week -2**	**Week 0**	**Week 2**	**Week 6**	**Week 12**	**Week 16**
**Case report form**	**A**	**B**	**C**	**C**	**C**	**C2**
Patient characteristics	X					
Eligibility criteria		X				
Informed consent	X					
Randomisation		X				
**Clinical measurements**						
Automated sphygmomanometer blood pressure, heart rate		X	X	X	X	
Height, waist circumference		X				
Weight		X			X	
Fasting lipids (total, LDL, HDL, triglycerides)	X		X		X	
Biochemistry (glucose, creatinine, CK, K+, Na+, ALT, AST)	X		X		X	
Serum ACE activity (subset of participants)					X	
**Other data collection**						
Medical history		X				
Current medications		X	X	X	X	
CV lifestyle interventions (smoking, alcohol, diet, exercise)		X			X	
Adverse events			X	X	X	X
Tolerability of trial drugs			X	X	X	
Pill count			X	X	X	
Abbreviations: LDL = low density lipoprotein, HDL = high density lipoprotein, K+ = potassium, Na+ =sodium, CK =creatine kinase, ALT = alanine aminotransferase, AST =aspartate transaminase, CV = cardiovascular						

11.1 Registration

The following procedures will be performed at registration:

1. Obtain written informed consent.
2. Arrange for fasting blood test (lipids and biochemistry).
3. Document baseline fasting blood test results.

11.2 Baseline visit

The following procedures will be performed at the baseline visit:

1. Record blood pressure, heart rate and calculate CV risk.
2. Assess participant eligibility according to trial inclusion and exclusion criteria.
3. Record demographic data, medical history and medication allergies.
4. Record all medication currently being taken by the participant.
5. Measure height, weight and waist circumference.
6. Record current lifestyle factors and interventions.

7. Randomise participant.

8. Provide participant with a 6 week treatment pack.

11.3 Week 2 and 6 follow-up visits

The following procedures will be performed at the follow-up visits 2 and 6 weeks after randomisation.

1. Record all medication currently being taken by the participant.

2. Record pill count (trial treatment).

3. Record adverse events that have occurred since the last trial visit.

4. Record if trial treatment has been withdrawn since the last trial visit and, if so, the main reason why.

5. Measure blood pressure and heart rate.

6. Arrange for fasting blood test (lipids and biochemistry) (2 week visit only).

7. Provide participant with a further 6 week treatment pack (6 week visit only).

8. Document blood results and review to identify abnormalities or sequential changes that may be relevant to trial treatment.

11.4 End of trial visit

The following procedures will be performed at the end of trial visit 12 weeks after randomisation.

1. Arrange for fasting blood test (lipid and biochemistry) and if relevant a urine salicylate test. (Within +/- 5 days of trial visit).

2. Record all medication currently being taken by the participant.

3. Record pill count (trial treatment).

4. Record current lifestyle interventions.

5. Record adverse events that have occurred since the last trial visit.

6. Record if trial treatment has been withdrawn since the last trial visit and, if so, the main reason why.

7. Measure blood pressure, heart rate and weight.

8. Document blood (and urine if relevant) results and review to identify abnormalities or sequential changes that may be relevant to post trial assessment.

9. Send thank you letter to participant with copy to participant's primary care practitioner (as locally relevant).

11.5 Post trial assessment

The following procedures will be performed at the post trial assessment, 16 weeks after randomisation (irrespective of whether or not the participant continues trial treatment for the duration of the treatment period).

1. Record side effects that have occurred since the end of trial visit.

11.6 End of trial

At the end of the trial when the data is unblinded send the participant and their primary care practitioner (as locally relevant) an end of trial letter.

12. Adverse event reporting

12.1 Adverse events

An adverse event (AE) is any untoward medical occurrence in a participant administered a pharmaceutical product and which does not necessarily have a causal relationship with this treatment. An AE can therefore be any unfavourable and unintended sign (including an abnormal laboratory finding), symptom, or disease temporally associated with the use of the trial treatment, whether or not related to the trial treatment.

All AEs must be recorded and described on the appropriate AE form (Form X).

Participants experiencing AEs that cause interruption or discontinuation of trial treatment, or those experiencing AEs that are present at the end of their participation in the trial should receive follow-up as appropriate by the trial investigator.

Side effects will be identified through the follow-up process at week 2, week 6, week 12 visits and week 16 assessment. Participants will be asked what side effects (if any) they have experienced and side effects will be classified into the following categories:

- Gastric irritation (stomach pain, heart burn)
- Gastrointestinal ulceration and/or haemorrhage (including black stool)
- Other haemorrhage (excluding traumatic)
- Increased bleeding tendency (e.g. easy bruising)
- Cough
- Light headed/dizziness/hypotension including related to change in posture
- Headache
- Diarrhoea
- Fatigue
- Abdominal pain
- Constipation
- Flatulence
- Muscle pain or weakness
- Hypoglycaemia
- Hyperglycaemia
- Other side effect (specified)

In addition any abnormalities presenting from laboratory tests will be reviewed at each follow-up visit.

The following notable side effects / laboratory abnormalities warrant particular attention and specific management as indicated in Table 3.

Table 3 Notable side effects / laboratory abnormalities and their management

Side effect / toxicity	Grade / level	Step 1 - Initial action	Step 2 - Management	* Step 3 -Follow-up
Gastric haemorrhage	Symptomatic, evident blood loss	Stop trial treatment	Clinical care according to local guidelines	Ensure stable haemoglobin; if not go to step 2
Dyspepsia (heartburn, stomach pain)	Moderate, persistent	Stop trial treatment	Clinical care according to local guidelines	Ensure resolution; if alternative explanation plausible – consider recommencing trial treatment
Cough	Troublesome, persistent	Stop trial treatment	Clinical care according to local guidelines	Ensure resolution; if alternative explanation plausible – consider recommencing trial treatment
Hypotension	Symptomatic, no other transient explanation (e.g. dehydration)	Stop trial treatment	Clinical care according to local guidelines	Ensure resolution; if transient cause resolved – consider recommencing trial treatment
Myopathy	Grade 1 – mild pain, not interfering with function, persistent	Check CK, consider stopping trial treatment		Ensure resolution; if transient cause resolved – consider recommencing trial treatment
Myopathy	Grade 2 – pain interfering with function, persistent	Stop trial treatment, check CK	Clinical care according to local guidelines	Ensure resolution; if alternative explanation plausible – consider recommencing trial treatment
Raised CK	Up to 5 times increase and no symptoms of myopathy<br><br>Elevated 5 times or more	Use clinical judgment<br><br>Stop trial treatment irrespective of symptoms	Investigate according to local guidelines	Ensure resolution
Raised liver enzymes (ALT, AST)	> 2-fold rise from baseline	Stop trial treatment	Investigate according to local guidelines	Ensure resolution; if alternative explanation plausible – consider recommencing trial treatment
Renal impairment	> 50% rise in creatinine from baseline	Stop trial treatment	Investigate according to local guidelines	Ensure resolution; if alternative explanation plausible – consider recommencing trial treatment

CK = creatine kinase

* Step 3; in all circumstances if side effect or toxicity has not resolved following Step 3 then review as in Step 2.

12.1.1 Serious adverse events

An event that is serious may require expeditious handling to comply with regulatory requirements.

A serious adverse event (SAE) or reaction is any event (whether or not related to the trial treatment) that:

- Results in death;
- Is life-threatening (defined as an event in which the participant was at risk of death at the time of the event; it does not refer to an event which hypothetically might have caused death if it were more severe);
- Requires inpatient hospitalisation or prolongation of existing hospitalisation;
- Results in persistent or significant disability/incapacity;
- Is a congenital anomaly or birth defect; or
- Other important medical event (defined as any event that is not immediately life threatening and does not result in death or hospitalisation but which may jeopardise the participant or may require intervention to prevent one of the outcomes listed as a SAE above e.g. intensive treatment in a emergency room or at home for allergic bronchospasm; blood dyscrasias or convulsions that do not result in hospitalisation; or drug dependency or drug abuse.

Potentially serious adverse events should be followed to resolution or stabilisation by the trial investigator, and reported as SAEs if they become serious (according to the definition above).

12.2 Reporting of serious adverse events and serious suspected adverse drug reactions

All AEs will be recorded on the Adverse Event Form (X), irrespective of whether serious or non-serious, or whether they are suspected to be due to trial treatment. If known, the name of the underlying illness or disorder (i.e. the diagnosis) should be recorded, rather than symptoms.

Timely and complete reporting of all AEs is necessary to identify events that are treatment related or potentially treatment related, thereby allowing: (1) a greater understanding of the overall safety profile of the treatment; (2) appropriate modification of trial protocols; and (3) adherence to regulatory requirements.

A Serious Suspected Adverse Drug Reaction (SSADR) is an SAE that is fatal or life-threatening *and* suspected to be related to trial treatment *and* unexpected (i.e. unlabelled - not reported in the clinical safety section of the Investigator Drug Brochure). All SSADRs require expeditious handling to comply with regulatory requirements. The investigator must notify the International Coordinating Centre immediately (within 24 hours of becoming aware of the event). The International Coordinating Centre will collate events and send to the Regional Coordinating Centre within 48 hours of notification by the investigator. The Regional Coordinating Centre will report all SSADRs to the appropriate Ethics Committee(s) and regulatory authorities and, if appropriate, notify primary care physicians. The Regional Coordinating Centre may be required to request supporting documentation to enable full reporting of the event. The International Coordinating Centre will report all SSADRs to the Steering Committee and Dr Reddy's Laboratories, whose responsibility it will be to update the Investigator Drug Brochure.

12.3 Adverse event reporting flow chart

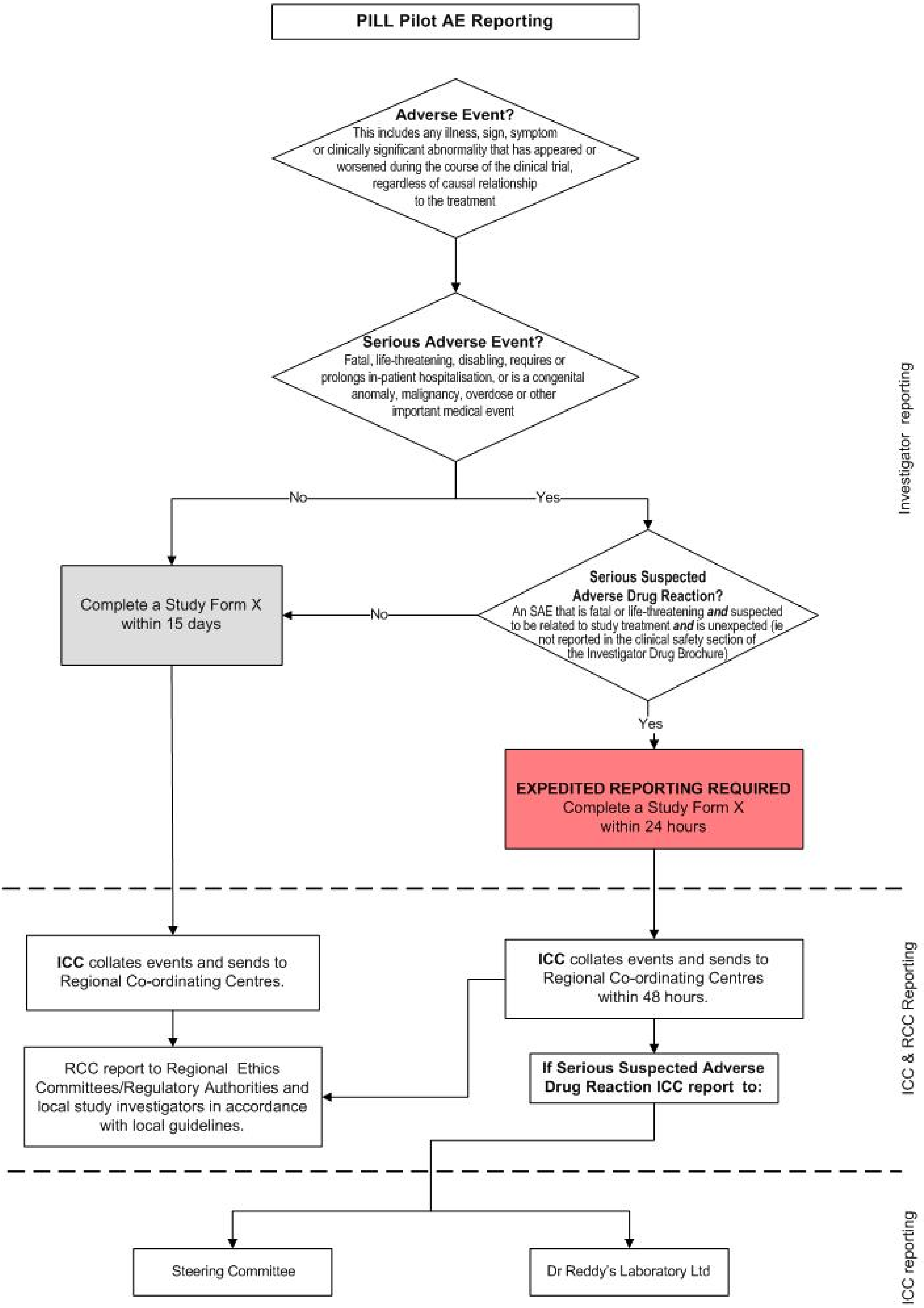

13. Monitoring

An independent trial monitor will be appointed by each Regional Coordinating Centre to monitor the trial in their region. The monitor will be permitted to visit each Local Trial Centre during the trial to ensure protocol, GCP and regulatory compliance.

Before the trial commences, the monitor will conduct an initiation visit with research staff at each site, consisting of education sessions with respect to the trial procedures (including adverse event reporting and inclusion/exclusion criteria), the nature of the trial, a review of the recruitment strategy, facilities and equipment, retention of essential documents and audit procedures. The monitor will also verify that trial documentation has been correctly distributed i.e. trial protocol and procedural manuals. Further education sessions will be conducted throughout the course of the trial where appropriate.

During the trial, the monitor will inspect every randomised participant's records to ensure their existence, that they meet the inclusion criteria and have provided informed consent, and that the polypill and placebo are being administered within the limits of the protocol.

The monitor will visit each centre no more than seven days after selection of the first participant and as frequently as deemed necessary by the monitor and Regional Coordinating Centre thereafter, with intervals of no more than two months.
Visits will be conducted to ensure that:
(1) Documentation is up-to-date [i.e. correct version of protocol and Manual of Procedures], record keeping meets the requirements specified in the protocol and complies with regulatory requirements;
(2) Electronic Case Report Forms (eCRFs) have been completed correctly and entries correspond to data within appropriate source documents;
(3) The site, facilities and materials used in the trial are adequate for their purpose and members of the trial team possess suitable education, training and experience to perform their functions in the trial;
(4) Polypill/placebo supply records are in order, the polypills/placebos are being stored appropriately and are not being used beyond the expiry dates, and that the handling of returned and/or unused polypill/placebo complies with trial procedures.

13.1 Close-out visit

Subsequent to the final participant completing their follow-up period and prior to database lock, a close-out visit will be conducted by the trial site monitor. The purpose of this visit is to ensure that all activities required for close-out – i.e. resolution of outstanding data queries or missing data, verification of archiving capability, destruction of unused trial treatment and end of trial notification - are complete and essential documents have been appropriately filed.

13.2 Audit

An audit or inspection may be performed at any stage before, during and after the trial by the sponsor, local or national regulatory authorities. In the event of an audit or inspection, the investigator must grant the following access to members of the audit or inspection team:

- To inspect the trial site, materials and facilities
- To meet all trial team members
- To access trial data, source documents and any other documents deemed relevant by the audit or inspection team.

If an investigator is informed of an impending audit or inspection the Regional Coordinating Centre and International Coordinating Centre should be informed immediately.

The monitoring requirements are also specified in the Monitoring Manual.

14. Trial treatment

14.1 Manufacture

Dr Reddy's Laboratories Ltd will be responsible for the quality of the trial treatment. The polypill and placebo will be manufactured to Good Manufacturing Practice (GMP) standards in a United States Federal Drug Agency approved formulation unit.

14.2 Identification

Dr Reddy's Laboratories Ltd will supply the polypill (aspirin 75mg, simvastatin 20mg, hydrochlorothiazide 12.5mg lisinopril 10mg) and matched placebo in 2-week calendar packs.

14.3 Packaging and labelling

The polypill and placebo will be packaged and labelled in compliance with GMP standards and according to the requirements of the regulatory authorities in each of the participating countries.

14.4 Handling and dispensing

Regional Coordinating Centres will be responsible for polypill and placebo importation into their countries, possibly via a trial wholesaler, according to the requirements of the regulatory authorities in each of the participating countries. Regional Coordinating Centres will be responsible for ensuring that Local Trial Centres have an adequate supply of the polypill and placebo.

Local Trial Centres will dispense the polypill and placebo to participants by authorised personnel. Upon dispensing the polypill or placebo, it is the investigators responsibility to ensure that the participant number and date dispensed are recorded.

14.5 Trial treatment supply records at Regional Coordinating Centres

The Regional Coordinating Centres will track and document supplies of polypill and placebo at each stage of the trial in their region using information from the trial wholesaler, where applicable, and investigators. Records or logs will include:

- Amount received from Dr Reddy's Limited and imported into each participating country by the trial wholesalers.
- Amount transferred to each Local Trial Centre.
- Label ID number or batch number.
- Dates of treatment inventory movement.
- Unique participant identifier (when allocated).
- Amount dispensed to each participant.
- Initials of person responsible for each treatment inventory entry.

14.6 Trial completion or termination

Upon completion or termination of the trial (after close out and before archiving), all unused dispensed polypill and placebo must be returned to Regional Coordinating Centres (or a designated trial wholesaler) via Local Trial Centres. All polypill and placebo returns must be accompanied by the appropriate documentation and be clearly identified by trial name and Local Trial Centre number on the outermost shipping container. Returned supplies should be in the original containers. Empty containers should not be returned. Regional Coordinating Centres will ensure the destruction of all returned dispensed polypill and placebo.

15. Trial power and planned analyses

Knowing LDL reduction, BP reduction and tolerance is essential to estimate risk reductions for the main trial, and hence design that trial appropriately. The main results from the PILL pilot analyses on change in blood pressure and cholesterol will be taken and modelled together with the expected effect of aspirin to provide the proportional reduction in cardiovascular events. This will involve combining trial evidence on the known impacts on CVD for given changes in blood pressure, cholesterol, and for taking anti-platelet therapy. Relative risk reductions will be calculated separately based on the observed change in blood pressure and cholesterol overall and then combined under a multiplicative model together with the RR for aspirin. Additional exploratory analyses may also be carried out based on varying assumptions behind these models.

15.1 Sample size

Approximately 400 participants will provide 85% power at 2p=0.05 to detect a 0.25 mmol/l difference in LDL cholesterol and 80% power to detect a 4 mmHg difference in systolic blood pressure between the intervention and control groups, assuming standard deviations around the change from baseline score of 0.8 mmol/l and 14 mmHg respectively. Hence a reduction in SBP will be assessed with a confidence interval width of about 5.5 mmHg (eg mean 10 mmHg, 95% CI 7.3-12.7 mmHg) and a cholesterol reduction will be assessed with a confidence interval of about 0.3 mmol/l (eg mean 1.1 mmol/l, 95% CI 0.94 - 1.26 mmol/). This number of participants will provide 80% power to detect a 10% absolute difference in tolerability (e.g. 5% tolerability in the placebo group versus 15% in the intervention). Confidence interval widths for a range of sample sizes are shown below:

Total sample size	250	350	450	550	650
Width of 95% CI for SBP reduction (mmHg)*	6.9	5.9	5.2	4.7	4.3
Width of 95% CI for LDL reduction (mmol/L)**	0.40	0.34	0.30	0.27	0.25

- assumes SD around reduction in SBP of 14 mmHg

** assumes SD around reduction in LDL of 0.8 mmol/L

15.2 Statistical analysis

All statistical analyses will be performed using SAS version 9.1.3 (SAS Institute Inc. Cary NC), R[1] and SPLUS[2]. All statistical tests will be two-tailed and a 5% significance level

[1] R-collaboration. http://www.r-project.org/.
[2] Becker, R., J. Chambers, and A. Wilks, *The New S Language: A Programming Environment for Data Analysis and Graphics*. 1991.

maintained throughout the analyses. All treatment evaluations will be performed on the principle of 'intention to treat' unless otherwise specified.

The full Statistical Analysis Plan comprehensively documents all planned statistical analysis in accordance with the principles below.

15.2.1 Baseline characteristics

Baseline comparability of the intervention and control group will be assessed via descriptive analyses, in terms of age, sex, ethnicity, smoking status, medical history, blood pressure and cholesterol.

15.2.2 Effects of treatment

Means of changes in blood pressure and cholesterol from baseline to 12 weeks between polypill and placebo groups will be compared using a 2 sample t-test. Adjusted analyses will be carried out by including the stratification factors in an analysis of covariance regression model with change in blood pressure and cholesterol as the dependent variable.

15.2.3 Safety and tolerability of the polypill

<u>Tolerability</u>

The proportions that withdraw from trial treatment (tolerability) at 12 weeks between polypill and placebo groups will be compared using the chi-squared test.

<u>Incidence of adverse events</u>

Incidence of all adverse events will be summarized, with specific categorisation and analysis of SAEs and deaths, and the incidence rate ratios comparing polypill with placebo will be calculated.

Total number of AEs, number of patients with at least one AE, and number of patients under each organ class and preferred term will be collated and analysed and ultimately summarised. Patients may have more than one AE per system organ class and preferred term. At each level of patient summarisation, a patient will be counted once if he/she reports one or more events.

All adverse events will be listed with separate listings prepared for each of the following categories and AEs separately:

<u>Relationship of AEs to Trial Drug</u>

AEs will be summarized by relationship (unknown, unrelated, unlikely, possibly and probable). As an additional summary, possibly and probable will also be grouped into "related", while unrelated and unlikely to be related will be grouped into "not related".

Patients may have more than one AE per body system and preferred term. At each level of summarization, patients will be counted once for the most related event if they reported one or more events.

<u>Severity of AE</u>

AEs will be summarized by severity: mild, moderate, and severe. Patients may have more than one adverse event per system organ class and preferred term. At each level of patient

summarization, patients will be counted once for the most severe event if they reported one or more events. If the severity of an AE is missing, the severity will be reported as "missing".

Serious AEs

Serious AEs will be described separately.

AEs Leading to Treatment Discontinuation/Trial Termination

AEs leading to treatment discontinuation/trial termination will be listed separately.

Death

AEs with fatal outcome will be listed separately

15.2.4 Subgroup analyses

Subgroup analyses will be performed according to age, sex, ethnicity, country, baseline 5-year risk according to the Framingham risk score, smoking status and baseline levels of blood pressure and blood lipids.

15.2.5 Unplanned Analysis

In May 2009 The Steering Committee decided to conduct an interim analysis that was not scheduled at the time of trial initiation, in order to provide data to the Wellcome Trust who were making a decision whether to fund the main trial at a meeting on June 5. A decision to complete the trial as planned (with no alterations to follow-up or data collection) was made prior to the unblinding, and only those members presenting to Wellcome were to be unblinded.

16. Administrative section

16.1 Trial registration

The trial has been registered with the Australian New Zealand Clinical Trials Registry (ACTRN012607000099426).

16.2 Roles and responsibilities

16.2.1 Steering Committee

The Steering Committee is responsible for providing strategic guidance for the trial including developing and maintaining the trial design, statistical analysis, presentation and publication of results. It is also responsible for approval of the protocol. The Committee will meet at least twice a year via teleconference or face-to-face to review problems and issues raised by the Regional and International Coordinating Centres. Dr Reddy's Laboratories will not be represented on the Steering Committee.

Steering Committee members

Professor Anthony Rodgers, Chair
Clinical Trials Research Unit
University of Auckland
New Zealand

Dr Natasha Rafter
Clinical Trials Research Unit
University of Auckland
New Zealand

Stephen Vander Hoorn
Clinical Trials Research Unit
University of Auckland
New Zealand

Professor Rod Jackson
Section of Epidemiology & Biostatistics
School of Population Health
University of Auckland
New Zealand

Associate Professor Bruce Neal
The George Institute of International Health
University of Sydney
Australia

Dr Anushka Patel
The George Institute of International Health
University of Sydney
Australia

Professor Srinath Reddy
All India Institute for Medical Sciences
New Delhi
India

Professor P Krishnam Raju
CARE Hospital,
Hyderabad
India

Professor Neil Poulter
National Heart and Lung Institute
Imperial College, London
United Kingdom

Professor Simon Thom
National Heart and Lung Institute
Imperial College, London
United Kingdom

Professor Richard Grimm
Berman Center for Outcomes and Clinical Research
Minneapolis Medical Research Foundation
USA

Professor Jim Neaton
Division of Biostatistics
University of Minnesota
USA

Professor Otavio Berwanger
Hospital do Coração (Cardiac Hospital)
Sao Paulo
Brazil

Professor Rick Grobbee
Julius Center for Health Sciences and Primary Care,University Medical Center
Utrecht
The Netherlands

16.2.2 International Coordinating Centre

The International Coordinating Centre, based in the Clinical Trials Research Unit (CTRU) at the University of Auckland, reports to the Steering Committee and is responsible for the daily operation of the trial. The International Coordinating Centre will perform central coordination, including development of trial materials, liaison with Dr Reddy's Laboratories, data management, central monitoring system, data analysis and training.

International Coordinating Centre staff

Professor Anthony Rodgers
Principal Investigator

Angela Wadham
Project Manager

Stephen Vander Hoorn
Senior Biostatistician

Barry Gray
IT Manager

Rina Prasad
Senior Data Manager

16.2.3 Regional Coordinating Centres

Regional Coordination Centres will be set up in each country by the country representatives on the Steering Committee. Regional Coordinating Centres will recruit and train Local Trial Centres and are responsible for ensuring recruitment targets are met and that the trial complies with local ethics, legislative and regulatory requirements including reporting.

Regional Coordinating Centres

**Region**	**Centre**
Australia	The George Institute for International Health, The University of Sydney, Sydney, Australia
Brazil	Hospital do Coração (Cardiac Hospital), Sao Paulo, Brazil
India	The George Institute India, Hyderabad, India
New Zealand	Clinical Trials Research Unit, The University of Auckland, Auckland, New Zealand
The Netherlands	Julius Center for Health Sciences and Primary Care, University Medical Center, Utrecht, The Netherlands
UK	International Center for Circulatory Health, London, UK
USA	Berman Center for Outcomes and Clinical Research, Minneapolis Medical Research Foundation, Minneapolis, USA

16.2.4 Local Trial Centres

Local Trial Centres will be responsible for identifying potential participants, participant registration and randomisation, conduct of the baseline visit and 2, 6, 12 and 16 week follow-ups, data collection, and reviewing and resolving queries, according to the protocol and Manual of Procedures.

16.3 Training

It is essential that trial staff understand why certain procedures are required and why data are collected, as well as how to perform protocol-required procedures and collect the data. A variety of training formats and materials will be used for this trial to ensure that trial staff at all levels are familiar with the requirements of the trial. Due to the international scope of the project, a “train the trainer” model will be used for protocol education.

Start-up training sessions at the regional level, conducted by staff from the International Coordinating Centre and members of the Steering Committee will be held to train Regional Coordinating Centre staff. Training materials (e.g. overheads, slide sets) will be provided by the International Coordinating Centre for Regional Coordinating Centre staff to use in training the Local Trial Centres for which they are responsible. Case scenarios will be developed and sample case report forms (CRFs) will be completed to illustrate how data are documented for hypothetical trial participants.

If changes in protocol or procedures necessitate remedial training, this training may occur via teleconference or through regional meetings, depending on the scope of the changes. Staff at the Regional Coordinating Centres will have the responsibility of training new site personnel in the case of staff turnover.

A Manual of Procedures will be developed by the International Coordinating Centre, which will provide documentation for procedures of the trial that are beyond the scope of the protocol document. The Manual of Procedures will contain instructions on patient screening and randomization, scheduling visits, completion of CRFs, and reporting endpoints and adverse events. The Manual of Procedures will be augmented by compilations of Frequently Asked Questions (FAQs), which will be written by the International Coordinating Centre in collaboration with the Regional Coordinating Centres.

16.4 Adherence to the protocol

Except for changes to eliminate an immediate hazard to participants, the approved protocol will be followed as specified. Any significant protocol deviation will be reported on a Protocol Violation Form via the central web-based data collection system.

16.5 Protocol revisions

All substantive revisions will be discussed with, and approved by, the Steering Committee. Any changes to the protocol will result in an update to version number and date. Administrative changes will be discussed with, and approved by, the International Coordinating Centre or Principal Investigator.

If the revision is an administrative change (such as the addition or removal of members to the committees), a letter explaining the change(s) along with a copy of the amended pages of the protocol must be submitted to the relevant Ethics Committees by the relevant Regional Coordinating Centres for their information. No formal approval from the Ethics Committees is required prior to implementation of administrative changes.

If the revision requires a change to a procedural aspect of the trial (for example, a change to the inclusion criteria or planned trial visits), it is classified as an Amendment. In this case, the Amendment (including the updated pages of the protocol and any other affected documentation) must be submitted to all relevant Ethics Committees for review and approval **prior** to implementation. Documentation of approval, signed by the chairperson or designee of the relevant Ethics Committees, must be filed in the Trial Master File.

If an Amendment substantially alters the trial design or increases the potential risk to the subject:

(1) the consent form will be revised and submitted to the relevant Ethics Committees for review and approval;

(2) the revised form will be used to obtain consent from subjects currently enrolled in the trial if they are affected by the Amendment; and

(3) the new form will be used to obtain consent from new subjects prior to enrolment.

Amendments may also need to be submitted to appropriate national medicine regulatory authorities.

New versions of the approved protocol will be circulated to all persons involved in the conduct of the trial, with instructions to supercede any previous versions. Where a protocol amendment directly affects the participant, information explaining the change to the protocol must be made available to the participant.

16.6 Case report form procedures

CRFs will be completed at the time of the participant visit, via the central web-based data collection system. Participants are to be identified by initials, birth date and registration number. All computerized forms will be electronically signed by the investigator and all changes made following the electronic signing will have an electronic audit trail stamped with a user name and date. Validations will be set up to help identify keying errors and out-of-range or unexpected data. The investigator will be responsible for reviewing the 'queries' that arise from these validations and for resolving them as appropriate.

Monitoring of a sample of the data will be conducted to ensure accurate transcription of the source data to the electronic forms.

16.7 Data confidentiality and security

Data will be entered, stored and backed-up in a secure manner via the CTRU internet data management system. (Further details will be provided in the Manual of Procedures)

16.8 Periodic reports

The International Coordinating Centre will provide regular reports of the progress, or completion, termination or discontinuation of the trial to the Steering Committee, Regional Coordinating Centres and Dr Reddy's Laboratories Limited.

16.9 Records retention

Polypill and placebo supply records will be retained by the RCC. Copies of eCRFs (or electronic files) will be retained by the CTRU (or an approved archivist) for the maximum period required by relevant legislation and/or regulations, as advised by the Regional Coordinating Centres.

16.10 Insurance

Regional Coordinating Centres (RCCs) will be required to determine the insurance requirements of their country. Dr Reddy's Laboratories Limited will provide each RCC with Clinical Trials Insurance cover.

16.11 Ownership of data and publication

Individual trial data remains the property of trial participants. The CTRU has responsibility for storage, protection and retrieval of trial data. The Steering Committee has responsibility for the safe guardianship and use of the data.

All publications will be approved by the Steering Committee, the membership of which will be named on all reports. The trial team and participants will be acknowledged in the final report, and in publications resulting from the trial.

Further documentation of this process will be made in a Data Use Policy paper (to be developed).

16.12 Funding

The following funding sources are secured or anticipated:

- Dr Reddy's Laboratories Limited - manufacture and supply of polypill and placebo, and funding for investigators meetings
- Health Research Council of New Zealand (International Investment Opportunities Fund) - recruitment and follow-up of participants in New Zealand.
- National Heart Foundation of New Zealand - Research Fellow at the International Coordinating Centre.
- National Health & Medical Research Council - recruitment and follow-up of participants in Australia.
- Wellcome Trust – international co-ordination, recruitment and follow-up of participants, bioequivalence testing of the polypill.
- British Heart Foundation - recruitment and follow-up of participants in the United Kingdom.

Other sources of funding are being sought to enable recruitment and follow-up in other participating countries.

References

1. World Health Organization. *The World Health Report 2002. Reducing risks, promoting healthy life.* Geneva: WHO, 2002.
2. Law M, Wald N. Risk factor thresholds: their existence under scrutiny. *BMJ* 2002;324:1570-6.
3. New Zealand Guidelines Group. Best practice evidence-based guideline. The assessment and management of cardiovascular risk. December 2003 ed: New Zealand Guidelines Group, 2003.
4. Neaton JD, Blackburn H, Jacobs D. Serum cholesterol level and mortality findings for men screened in the Multiple Risk Factor Intervention Trial. *Archives of Internal Medicine* 1992;152:1490-500.
5. Yusuf S, Sleight P, Pogue J, Bosch J, Davies R, Dagenais G. Effects of an angiotensin-converting-enzyme inhibitor, ramipril, on cardiovascular events in high-risk patients. The Heart Outcomes Prevention Evaluation Study Investigators. *New England Journal of Medicine* 2000;342:145-53.
6. PROGRESS Collaborative Group. Randomised trial of a perindopril-based blood-pressure-lowering regimen among 6105 individuals with previous stroke or transient ischaemic attack. *Lancet* 2001;358:1033-41.
7. McNeil J, Peeters A, Liew D, Lim S, Vos T. A model for predicting the future incidence of coronary heart disease within percentiles of coronary heart disease risk. *Journal of cardiovascular risk* 2001;8:31-37.
8. Rafter N, Connor J, Hall J, Jackson R, Martin I, Parag V, et al. Cardiovascular medications in primary care: treatment gaps and targeting by absolute risk. *New Zealand Medical Journal* 2005;118(1223).
9. Referred Services Advisory Group. Referred services management: building towards equity, quality and better health outcomes. Report of the Referred Services Advisory Group to the Ministry of Health, October 2002. *www.moh.govt.nz* 2002.
10. Antithrombotic Trialists' Collaboration. Collaborative meta-analysis of randomised trials of antiplatelet therapy for prevention of death, myocardial infarction, and stroke in high risk patients. *British Medical Journal* 2002;324:71-86.
11. Blood Pressure Lowering Treatment Trialists' Collaboration. Effects of different blood-pressure-lowering regimens on major cardiovascular events: results of prospectively-designed overviews of randomised trials. *Lancet* 2003;362:1527-35.
12. Law MR, Wald NJ, Morris JK, Jordan RE. Value of low dose combination treatment with blood pressure lowering drugs: analysis of 354 randomised trials. *BMJ* 2003;326(7404):1427-31.
13. The ALLHAT Officers and Coordinators for the ALLHAT Collaborative Research Group. Major outcomes in high-risk hypertensive patients randomized to angiotensin-converting enzyme Inhibitor or calcium channel blocker vs diuretic The Antihypertensive and Lipid-Lowering Treatment to Prevent Heart Attack Trial (ALLHAT). *Journal of the American Medical Association* 2002;288:2981-97.
14. Wald N, Law M. A strategy to reduce cardiovascular disease by more than 80%. *British Medical Journal* 2003;326:1419-1424.
15. ICH Expert Working Group. ICH harmonised tripartite guideline for good clinical practice EG(R1): ICH, 1996.

Appendix 1 –– Informed consent procedures

Written, informed consent MUST be obtained from all participants prior to inclusion in the trial. The signed consent will be filed in the participant's trial file. For written consent to be valid the participant must be suitably informed of the trial so that they can make an independent choice about whether to participate. The participant should receive a copy of the consent form at the trial visit. Issues to be covered in the information sheet should be reviewed carefully with each participant. Do not assume that every person has read the information sheet or that they can read. The consent form should be signed and dated by the participant and by the person conducting the consent process (the Principal Investigator or an approved designated person). The potential participant should have details regarding:

- The purpose of the trial.
- An explanation of who the researchers are.
- An explanation of why the participant qualifies for the trial.
- The type of participants studied and the number likely to be involved.
- The length of the trial.
- Any procedures, assessment or interventions to be conducted.
- The potential risks/benefits to the person.

The potential participant should be informed that:

- The supply of information by the participant is entirely voluntary.
- The participant may refuse to answer any of the questions or refuse any of the clinical examinations. They do not have to give a reason for doing so.
- All participants have the right to access their data and/or to remove it from the trial.
- All participants have the right to have questions answered.
- A person outside of the trial is available to be contacted should they have any concerns i.e. a health advocate.

The participant should be aware that:

- Personal information will be collected about them but that this information will be kept strictly confidential.
- Data relating to the participant will be identified only by the participant's trial registration number, date of birth and initials.
- The information will be kept in a secure environment at the Local Trial Centre and/or in a locked room at the Regional Coordinating Centre and/or the International Coordination Centre.
- All computerised information will be password protected on a computer.
- Only the trial team and sponsor, local or national regulatory auditors / inspectors (if applicable) will have access to this data.
- All information will be published or presented in a way that no individual can be identified.
- The participant's primary care practitioner will be informed of their participation in the trial and the trial results when complete (as locally relevant).

Appendix 2 – ICH GCP principles

Principles of the International Conference on Harmonisation Tripartite Guideline for Good Clinical Practice[15]:

1. Clinical trials should be conducted in accordance with the ethical principles that have their origin in the Declaration of Helsinki, and that are consistent with GCP and the applicable regulatory requirement(s).

2. Before a trial is initiated, foreseeable risks and inconveniences should be weighed against the anticipated benefit for the individual trial subject and society. A trial should be initiated and continued only if the anticipated benefits justify the risks.

3. The rights, safety and well-being of the trial subjects are the most important considerations and should prevail over interests of science and society.

4. The available nonclinical and clinical information on an investigational product should be adequate to support the proposed clinical trial.

5. Clinical trials should be scientifically sound and described in a clear, detailed protocol.

6. A trial should be conducted in compliance with the protocol that has received prior institutional review board (IRB)/independent ethics committee (IEC) approval/favourable opinion.

7. The medical care given to, and medical decisions made on behalf of, subjects should always be the responsibility of a qualified physician or, when appropriate, of a qualified dentist.

8. Each individual involved in conducting a trial should be qualified by education, training and experience to perform his or her respective task(s).

9. Freely given informed consent should be obtained from every subject prior to clinical trial participation.

10. All clinical trial information should be recorded, handled and stored in a way that allows its accurate reporting, interpretation and verification.

11. The confidentiality of records that could identify subjects should be protected, respecting the privacy and confidentiality rules in accordance with the applicable regulatory requirement(s).

12. Investigational products should be manufactured, handled and stored in accordance with applicable good manufacturing practice (GMP). They should be used in accordance with the approved protocol.

13. Systems with procedures that assure the quality of every aspect of the trial should be implemented.

Appendix 3 – Protocol Signature Page

By signing below, I confirm that I have received, read and understood the protocol Version 7.0. dated 29 June 2009, for the PILL (Programme to Improve Life and Longevity) pilot trial.

I agree to follow the protocol and attachments, and provide the necessary assurance that this trial will be conducted according to all stipulations of the protocol, including all statements regarding confidentiality, and according to local legal and regulatory requirements and to the principles outlined in the International Conference on Harmonisation Tripartite Guideline for Good Clinical Practice (Appendix 2).

If other personnel under my supervision are involved in the trial I will provide and discuss the protocol with them to ensure that they are fully informed about the treatment and the trial.

I understand that the protocol may be revised at any time and I undertake to ensure the most current version is adhered to at all times.

I understand that the trial may be terminated or enrolment suspended at any time if it becomes necessary to protect the best interests of the trial participants.

Signature: ______________________________

Investigator Name: ______________________________

Trial Centre: ______________________________

Date: ______________________________
